# Magnetic structure determination and refinement using *FullProf*

**DOI:** 10.1107/S2052520625003944

**Published:** 2025-05-28

**Authors:** Juan Rodriguez-Carvajal, Javier Gonzalez-Platas, Nebil A. Katcho

**Affiliations:** a Diffraction and Computing for Science Groups Institut Laue Langevin (ILL) 71 Avenue des Martyrs, CS 20156 38042Grenoble Cedex 9 France; b Departamento de Física. Instituto Universitario de Estudios Avanzados en Física Atómica, Molecular y Fotónica (IUDEA), MALTA Consolider Team Universidad de La Laguna Avenida Astrofísico Francisco Sánchez s/n La Laguna TenerifeE-38204 Spain; Brigham Young University, USA

**Keywords:** magnetic (super)space groups, incommensurate magnetic structures, simulated annealing, magnetic crystallography, symmetry modes

## Abstract

The study of magnetic structures from a crystallographic perspective remains a niche field, largely confined to physical crystallography and microscopic magnetism. Unlike general or superspace crystallography, magnetic structure analysis lacks standardization, primarily due to the small research community and the reliance on specialized experimental techniques like neutron diffraction. However, emerging topics in condensed matter physics, such as topological materials, multiferroics, and skyrmions, have heightened the importance of understanding magnetic ordering. As a result, the analysis of magnetic neutron scattering data is becoming increasingly relevant, requiring unified methodologies. Since 2011, the IUCr Commission on Magnetic Structures has been working to improve the situation. This paper reviews the most commonly used methods for describing magnetic structures and highlights the capabilities of the *FullProf Suite* for analysing magnetic neutron diffraction data.

## Abstract 2

A description of the *FullProf Suite* of programs for magnetic structure determination is given. Different types of magnetic structure factors are discussed, and links to examples and tutorials for using the programs are provided.

## 1. Introduction

In conventional crystallography, a highly effective way to describe electron and nucleus density is through the concept of atomicity. This approach simplifies the scattering density by representing atoms as well defined entities, essentially mass points or spheres, characterized by their chemical identity, spatial positions within the unit cell, and displacement parameters that account for dynamic or static deviations from equilibrium positions. The symmetry of periodic atomic arrangements is well captured by the 230 space group types in three dimensions (3D) (Bradley & Cracknell, 1972 ▸). However, more intricate atomic structures may require descriptions that extend beyond three dimensions, utilizing the superspace approach, where atoms are represented as atomic surfaces (Janssen & Janner, 2014 ▸). While incommensurate, composite, and quasi-crystalline structures constitute only a small subset of materials that can be described through conventional 3D crystallography, their complexity requires higher-dimensional analysis. The widespread success of crystallography is largely attributed to the ability to visualize atoms directly, appearing as small quasi-spherical spots in modern imaging techniques such as electron microscopy, atomic force microscopy, and tunnelling microscopy.

Magnetic crystallography is a specialized branch of crystallography focused on describing and determining magnetization density, or, in quantum mechanical terms, spin density, in solids. The classical representations used to depict magnetic scattering density are vectors, illustrated as arrows, which define the elementary magnetic moments (dipoles) of atoms with unpaired electrons. A magnetic structure refers to a specific, nearly static spatial arrangement of these magnetic moments (*solid* phase), which forms below the ordering temperature. Above this temperature, the system becomes disordered and transitions into the paramagnetic (*liquid*) state.

Magnetic structures are typically visualized as a set of arrows representing non-null magnetic dipoles in the atoms, with distinct magnitudes and orientations depending on the structure. However, direct visualization of elementary magnetic moments using a dedicated magnetic microscope remains unattainable. Current magnetic imaging techniques rely on detecting magnetic fields generated by atoms with unpaired electrons, which is only effective for ferromagnets or ferrimagnets at the mesoscopic scale. Unlike crystal structures, many magnetic structures are non-commensurate, meaning the periodicity of their magnetic moment orientations does not align with the underlying crystal lattice. This results from competing exchange interactions and anisotropy terms, often leading to frustration in many compounds.

All our knowledge of magnetic structures stems from the analysis and interpretation of magnetic neutron diffraction patterns. General papers on the experimental determination of magnetic structures and their mathematical description are available in the literature (Rossat-Mignod, 1987 ▸; Brown, 1986 ▸; Rodríguez-Carvajal & Bourée, 2012 ▸; Rodríguez-Carvajal & Villain, 2019 ▸).

Two distinct approaches have traditionally been used to describe the symmetry properties of commensurate magnetic structures: magnetic space groups (MSG), also known as Shubnikov groups (Opechowski & Guccione, 1965 ▸; Opechowski, 1986 ▸; Litvin, 2001 ▸, 2013 ▸), and group representation analysis (Bertaut, 1963 ▸, 1968 ▸, 1971 ▸, 1981 ▸; Izyumov & Naish, 1979 ▸; Izyumov *et al.*, 1979*a* ▸, 1979*b* ▸, 1979*c* ▸, 1991 ▸; Izyumov, 1980 ▸). As demonstrated in Rodriguez-Carvajal & Perez-Mato (2024 ▸), these approaches are complementary, and their combined application provides the most effective means of analysing magnetic structures.

For incommensurate magnetic structures, representation analysis (RA) can be applied in the same way as for commensurate structures. However, the most comprehensive approach involves combining RA with the principles of superspace crystallography (Janssen & Janner, 2014 ▸). This method incorporates the spin reversal operator, which is a symmetry operator of the paramagnetic state, enabling the use of magnetic superspace groups (MSSG) (Pérez-Mato *et al.*, 2012 ▸).

This paper focuses on the various approaches implemented in the programs of the *FullProf Suite* and their evolution over the past thirty years. The *FullProf Suite* has been developed by the present authors and their collaborators since the early 1990s. The first published description of the features available in *FullProf* for incommensurate magnetic structures was provided by Rodríguez-Carvajal (1993 ▸). This paper also introduced the *MagSAN* program, which was designed for the determination of commensurate magnetic structures using simulated annealing. *MagSAN* was later incorporated into the *FullProf* program for general crystal and magnetic structures. At the turn of the century, three additional programs, *MODY*, *SARA*h** and *BasIreps* (the latter included in the *FullProf Suite*) (Sikora *et al.*, 2004 ▸; Wills, 2000 ▸; Rodríguez-Carvajal, 1998 ▸), became available and were developed to assist in determining magnetic structures. These programs employ the RA method, popularized by Bertaut (1963 ▸, 1968 ▸), to generate basis vectors of irreducible representations (*irreps*), and they have become widespread and widely used by the condensed matter physics and chemistry communities for the study of magnetic structures.

The *FullProf* program was initially developed to meet the needs of the first of the present authors and was later made available to a wider community. As a result, it is not a black-box tool that guides users through data analysis with a predefined workflow. Instead, users have complete control over the input model, provided they understand the rules governing the input control file (hereafter referred to as the PCR file). Consequently, the learning curve may be steeper than for other software programs.

Over time, several features have been introduced to enhance the program’s usability. In particular, the graphical user interface (GUI) program *EdPCR* allows users to manage most of the options in *FullProf* without directly editing the PCR text file. This interface also supports a hybrid workflow, where users can manually edit the PCR file within the GUI, make adjustments by hand, and reload the modified file. *EdPCR* will automatically detect any changes and prompt the user to reload the file accordingly.

The *FullProf Suite* continues to evolve and is currently the most widely used software package for the analysis of magnetic structures from powder or single-crystal neutron diffraction data. Two of the most significant developments in *FullProf* over the past five years are the combined use of RA and MSG for commensurate magnetic structures and the capability to handle MSSGs.

In the following sections, we will explore the different methods used to describe magnetic structures in the literature and how they have been implemented in the *FullProf* program. While we will primarily focus on the application of the program to neutron powder diffraction (NPD), it is also applicable to the analysis of integrated intensities obtained from neutron single crystal diffraction (NSCD).

## 2. Earlier approach: basic description of commensurate magnetic structures in the *magnetic* unit cell

In crystallography, independent atoms are described by their chemical nature, their position within the unit cell, and a set of symmetry operators, common to all atoms, that define one of the 230 space group types. These operators take the form {*R*_*s*_|**t**_*s*_},*s* = 1…*M*, where *M* represents the general multiplicity (number of coset representatives with respect to the translation group) of the space group, *R_s_* is a proper or improper rotation matrix and **t***_s_* its associated primitive translation. The symmetry operators generate the remaining atoms within the unit cell, and this extends in three directions forming a periodic structure.

Although MSGs were not fully tabulated in the early stages of their development, this approach closely resembled that of conventional crystallography and was also adopted in certain publications. An MSG contains operators of the form {*R*_*s*_, δ_*s*_|**t**_*s*_},*s* = 1…*M*, where the symbol *δ_s_* (called signature) is equal to −1 (indicating a primed operator) or +1, depending on whether the time-reversal operation is associated or not with the *s* operator. The action of these operators on atom positions is identical to that of ordinary crystallographic operators: 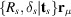 = 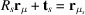.

The action of the operators on magnetic moments follows the transformation: 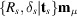 = 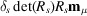 = 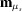.

Ignoring symmetry (except the translational symmetry), a complete list of all the atoms within the elementary unit cell, along with their fractional coordinates, atomic displacement parameters, and occupation probabilities, provides a full description of the crystal structure. For the sake of simplicity, thermal and occupancy parameters will be omitted in the following discussion. This list of chemical composition and coordinates is equivalent to a description in the space group number 1: *P*1.

Symmetry is of great importance as it reduces the number of free parameters that must be fitted from experimental data. Determining all atomic coordinates when describing the crystal structure in *P*1 is often challenging due to the limited number of observations and the correlations between parameters during refinement.

Describing a magnetic structure in *P*1 means that, in addition to specifying atomic coordinates, it is necessary to list the magnetic moments associated with each atom. It is important to note that the magnetic unit cell of a commensurate structure is generally a multiple of the crystallographic cell or may coincide with it. The components of the magnetic moments are expressed in Bohr magnetons, relative to a basis formed by unitary vectors along the conventional crystal basis *U* = (**a**/*a*, **b**/*b*,**c**/*c*) = (**e**_1_, **e**_2_, **e**_3_). This straightforward method of describing a magnetic structure, that we call the *P*1-approach, was commonly used in early literature and represents a special case of the approaches outlined below. The *P*1-approach was used together with constraints in magnetic moments that were deduced from the observed diffraction patterns and the particular expressions of the magnetic structure factor. No explicit reference to magnetic symmetry or RA is mentioned in the *P*1-approach. Examples of earlier magnetic structure determinations can be found in Corliss *et al.* (1956 ▸), Frazer (1958 ▸) and Scatturin *et al.* (1961 ▸).

## 3. Concept of propagation vector(s) for describing magnetic structures in the crystallographic unit cell:incommensurate magnetic structures

In the early stages of magnetic structure studies using neutron diffraction, it became evident that the positions of magnetic reflections in diffraction patterns could not always be described by a ‘magnetic unit cell’. This issue arose in the first observation of an incommensurate magnetic structure in MnAu_2_ [Herpin *et al.* (1959 ▸); see Rodríguez-Carvajal & Villain (2019 ▸) for further details on this discovery]. A straightforward mathematical formula was developed to describe the orientation of magnetic moments in real space, and this was applied to characterize the helical structure of the MnAu_2_ compound, introducing the concept of the propagation vector. The propagation vector concept is further generalized through the use of a Fourier series to determine the magnetic moment at any given point in the crystal:
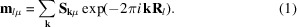
This defines the magnetic moment of the atom numbered μ within the primitive unit cell, with its origin at the position of the lattice vector **R***_l_*. The atomic position in the crystal given by **R**_*l*μ_ = **R***_l_* + **r**_μ_. The **k** vectors are defined in reciprocal space and are referred to as propagation vectors of the magnetic structure (where harmonics are treated as distinct **k** vectors). These vectors are confined to the first Brillouin zone (BZ), as the addition of a reciprocal lattice vector **H** does not alter the sum. Two propagation vectors are considered equivalent (**k**≡**k**′) if they differ by a reciprocal lattice vector. Any class of magnetic structure can be represented by the Fourier series (1).

Equation (1) may also be defined in a slightly different manner, which is common in the literature, particularly in the superspace approach. Instead of writing **R***_l_* in the argument of the exponential function, one can express it as **R**_*l*μ_ = **R***_l_* + **r**_μ_:
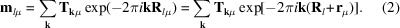
In this case, the Fourier coefficients, **T**_**k**μ_ are related to **S**_**k**μ_ by a phase factor **S**_**k**μ_= **T**_**k**μ_exp(−2π*i***kr**_μ_), which depends on the atomic positions within the unit cell. In the following discussion we will use the convention (1), as it offers certain advantages for commensurate structures, while convention (2) will be applied when working with magnetic superspace group symmetry. The general expression for the Fourier coefficients used in *FullProf* is given by
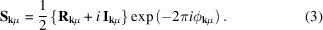
The phase factor is not strictly necessary, but it proves useful when constraints are applied to the components of **R**_**k**μ_ and **I**_**k**μ_ (for instance if |**R**| = |**I**| and **R**⊥**I**). When using the superspace approach for a single pair (**k**, −**k**) the convention (2) is used and the Fourier coefficients **T**_**k**μ_ are written as
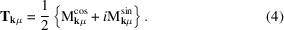
The magnetic Bragg reflections are indexed by the diffraction vectors **h** = **H** + **k**. Reflections where **k** ≠ 0 are referred to as *satellite* reflections, while those with **h** = **H** are known as *fundamental* reflections. Under the convention (1), the Fourier coefficients are identical to the magnetic moments when there is a single term in the sum and **k** = ½**H**.

Within *FullProf*, it is possible to work directly with the Fourier coefficients **S**_**k**μ_ utilizing all magnetic atoms within the unit cell, thereby extending the *P*1-approach to complex incommensurate structures.

## 4. RA as implemented in *BasIreps*

The initial implementation of symmetry constraints in *FullProf* used RA via the *BasIreps* program. The theoretical concepts underpinning this program can be found in section 7 of Rodríguez-Carvajal & Bourée (2012 ▸). The *BasIreps* program (Rodríguez-Carvajal, 1998 ▸) is capable of calculating the star of the propagation vector and algorithmically generating the *irreps* (small *irreps*) of the propagation vector group *G***_k_** (also known as the ‘little group’). The group *G***_k_** consists of the operators in the space group *G* that leave the propagation vector invariant (as defined in the previous section). *BasIreps* employs a subroutine from the *KAREP* program (Hovestreydt *et al.*, 1992 ▸) to compute the small *irreps* from which the full representations of the space group *G* can be derived, through the star of **k**, via the induction formula [see equation 30 in Rodríguez-Carvajal & Bourée (2012 ▸)]. In the simplest case of a single pair (**k**, −**k**) of an incommensurate structure, both arms of the star must be taken into account, and the *irreps* will have at least dimension 2. The symmetry operators present in the paramagnetic group transforming **k** into −**k** must be incorporated into the list of relevant operators (forming the extended little group *G***_k_**_,−**k**_); otherwise, neglecting these symmetries would lead to an unnecessary increase in the number of parameters required to describe the magnetic structure. *BasIreps* can also directly read the *irreps* of *G***_k_**_,−**k**_ (or the full *irreps* of *G*, as detailed below) from the database provided by Stokes *et al.* (2013 ▸). The basis vectors of the *irreps* are related to the Fourier coefficients (3) by the linear combination
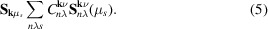
The index *s* enumerates the different atoms of the orbit of the particular site μ, obtained by applying the symmetry operators {*R_s_*|**t**_*s*_} of *G***_k_** (or *G***_k_**_,−**k**_) to the vector position of the first representative of site μ: **r***_μ_* = **r***_μ_*_1_. The vectors 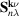 correspond to the atomic components of the basis vectors of the *irrep* Γ_ν_ and are expressed in the unitary basis *U* of the paramagnetic crystal structure. When the basis vectors are properly normalized, the coefficients 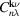 have units of Bohr magnetons. The index *n* ranges from 1 to *n*_ν_, where *n*_ν_ represents the number of times the *irrep* Γ_ν_ appears in the reducible magnetic representation Γ_mag_. Additionally λ ranges from 1 to dim(Γ_ν_), and these values can be computed using the *BasIreps* program. If more than one *irrep* contributes to the overall magnetic order, an additional summation over representations (ν) must be considered. For commensurate magnetic structures, the coefficients must be real, whereas for cases where **k** lies within the interior of the BZ, they may be complex.

**Figure 1:**
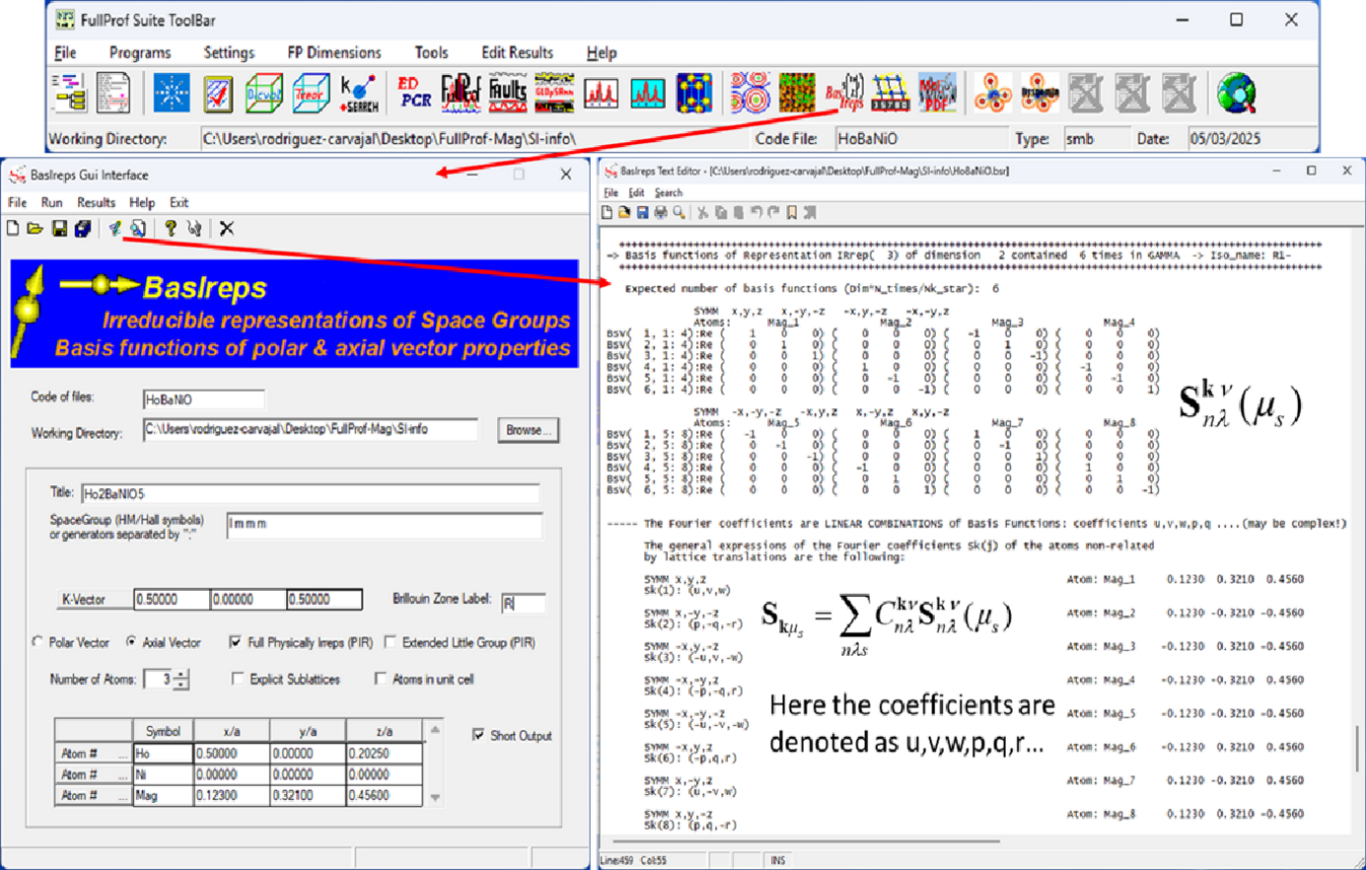
Layout of the *FullProf Suite* toolbar and *BasIreps* illustrating the results of a calculation, corresponding to the full star (two arms) of the propagation vector **k** = (1/2, 0, 1/2), located at the *R*-point in the BZ of *Immm*. The full *irreps* are 2D, and the generated basis vectors, along with their corresponding coefficients for the content of the primitive cell of the paramagnetic space group *Immm.*1, are displayed on the right side of the figure. In this case, the result corresponds to the MSG *Cmma*.1′_a_ (in UNI notation, BNS: *C*_a_*mma*) for the *irrep* associated with the order parameter direction (*a*, −*a*). An atom in a general position in *Immm.*1′ is consequently split into two independent positions in *Cmma*.1′_a_. The SYMM operators and Sk(s) values are referenced to the basis of the paramagnetic group.

As mentioned earlier, the standard function of *BasIreps* is to calculate the representations and basis vectors of the little group *G***_k_**. However, the current version of the program can also access the database of complete representation (the full star of **k**) of the space group *G*, using the database of physically irreducible representations provided by Stokes *et al.* (2013 ▸). This allows the calculation of basis vectors using projection operator formulas [see equation 48 of Rodríguez-Carvajal & Bourée (2012 ▸)] without explicitly controlling the direction of the order parameter. For *irreps* of dimension higher than 1, this approach may yield more 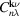 coefficients than strictly necessary, requiring the user to manually select the order parameter. While this method can accommodate any kind of magnetic structure, it may be cumbersome for users unfamiliar with the formalism. An example of *BasIreps* in use is illustrated in Fig. 1 ▸.

The current version of *BasIreps* generates two files: one containing the complete information (with a portion displayed in Fig. 1 ▸), and another with formatted sections that can be directly copied into the PCR file for data processing. The functionality of *BasIreps* is similar to that of *SARAh* (Wills, 2025 ▸), although the latter features a more user-friendly interface for integration with *FullProf*, making it more suitable for beginners. Examples of *BasIreps* applications in magnetic structure determination can be found in the publicly available tutorials recently uploaded to the *FullProf Suite* website, as referenced in the supporting information (SI). In the SI document we have created three sections that will be referred hereafter as SI-1, SI-2 and SI-3.

## 5. MSG utilities in the *FullProf Suite*

Information and calculations using MSGs can be found on the Bilbao Crystallographic Server (BCS) (https://www.cryst.ehu.es; Aroyo *et al.*, 2006*a* ▸, 2006*b* ▸, 2011 ▸) and on the *ISOTROPY* website (https://iso.byu.edu/iso/isotropy.php; Stokes *et al.*, 2023 ▸). Traditionally, two different notation systems have been used in the literature: Opechowski–Guccione (OG) (Opechowski & Guccione, 1965 ▸) and Belov–Neronova–Smirnova (BNS) (Belov *et al.*, 1957 ▸). Both systems have their own advantages and limitations. To address this, Campbell *et al.* (2022 ▸, 2024 ▸) have proposed unified symbols (UNI), which integrate a modified BNS symbol in a BNS setting while incorporating key information from the OG symbol. The UNI system is intended to gradually replace both BNS and OG notations for the description of commensurate magnetic structures in their standard setting.

**Figure 2:**
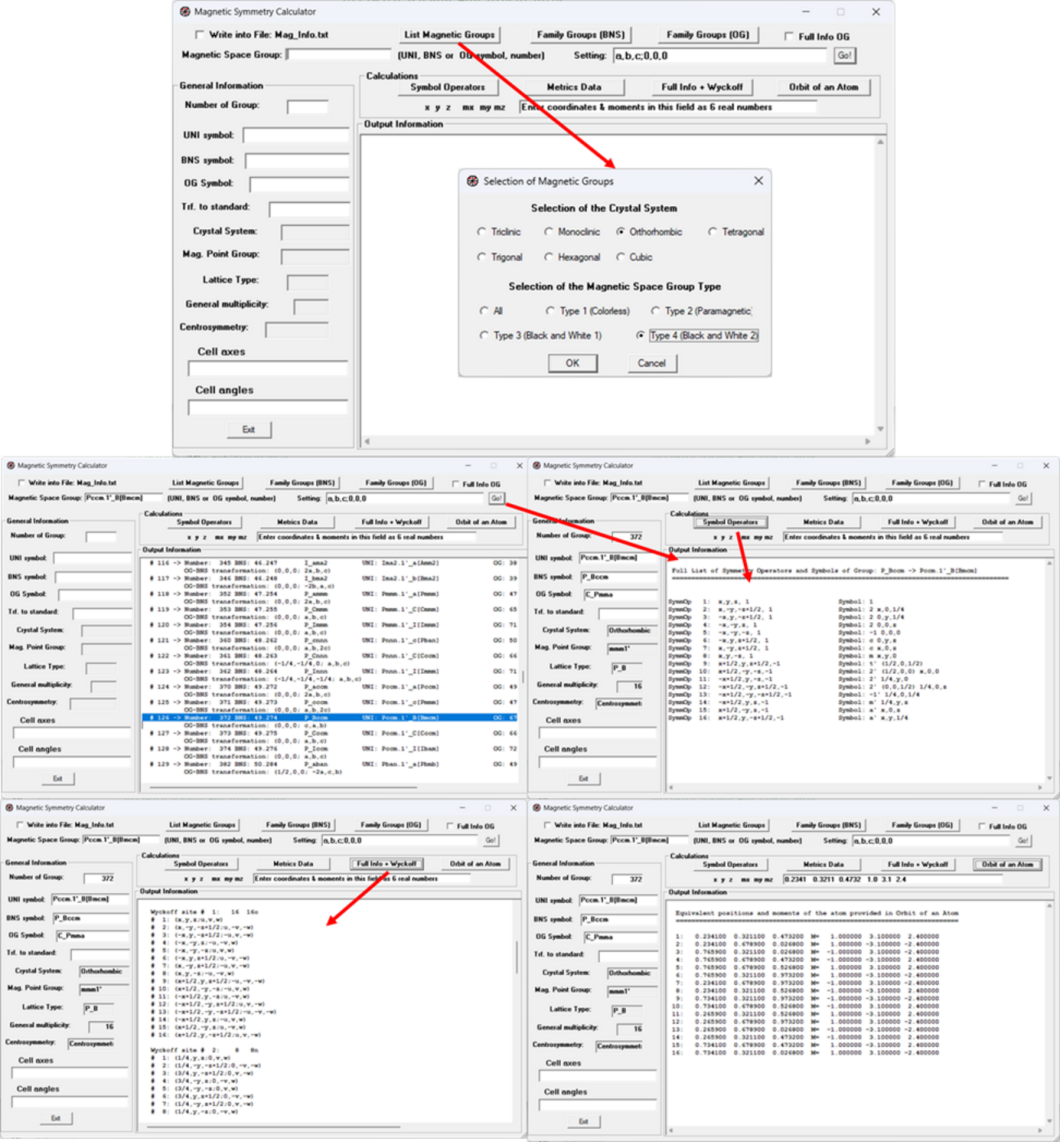
Layout of the magnetic crystallographic calculator. This application operates by selecting a MSG, entering its symbol (UNI, BNS or OG) into the designated field, and clicking on the Go! button. Once this is done, various calculations are performed by clicking the corresponding buttons, with results displayed in the output information area. The output can then be copied and pasted into other files as needed.

**Figure 3:**
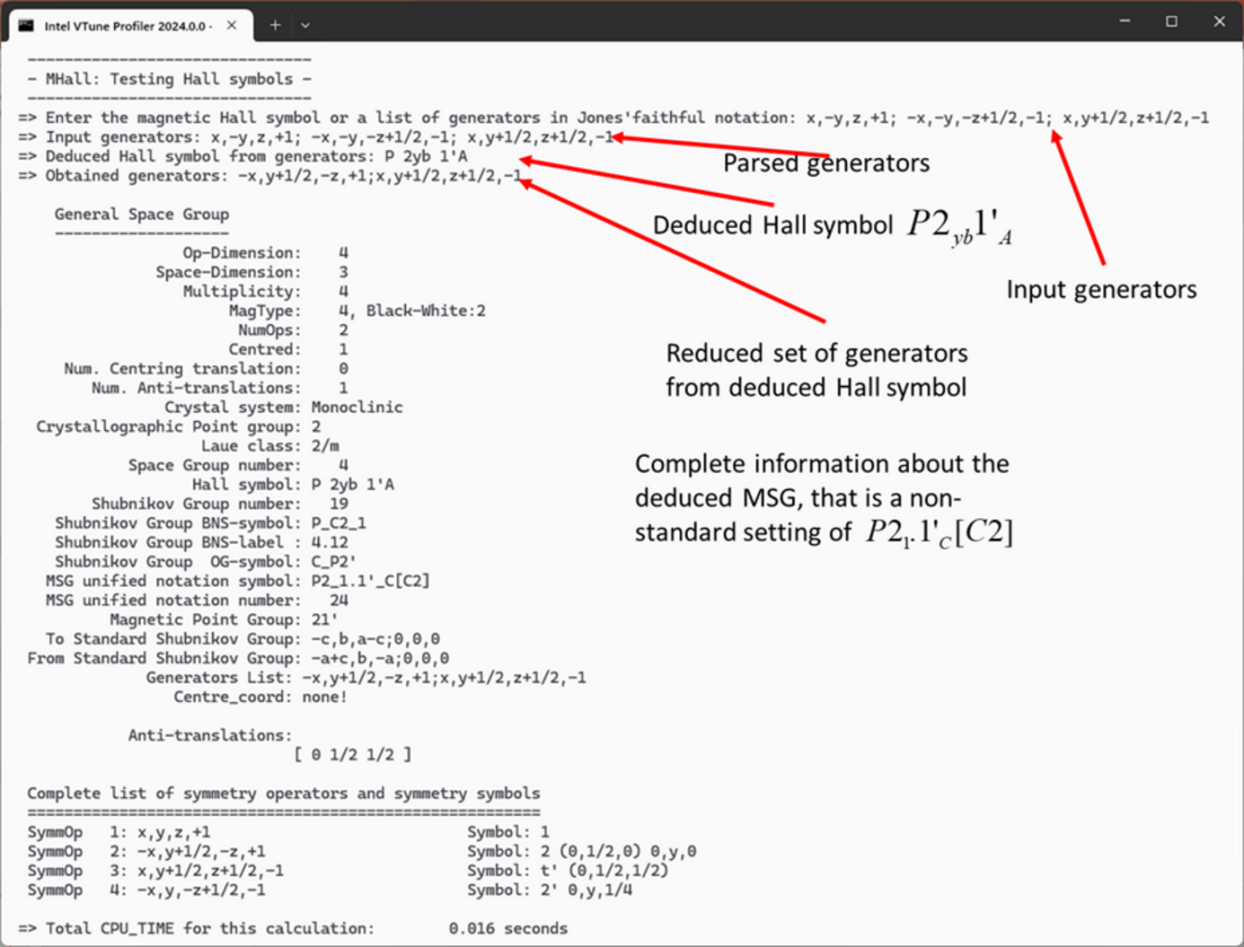
Example of calculation with the console program *MHall*.

González-Platas *et al.* (2021 ▸) introduced another type of symbol aimed at unambiguously defining the generators of the MSG represented in the symbol. The Hall symbols are constructed by applying a set of specific rules outlined in the aforementioned reference. From these symbols, it is straightforward to deduce the explicit form of the matrix, signature, and translation vectors of the generators. These symbols are not intended to replace the BNS, OG, or UNI notations; rather, they serve as a practical tool for generating the complete set of operators of an MSG by parsing the symbol in any kind of setting. The *FullProf Suite* provides two dedicated applications for working with MSGs:

(1) Magnetic symmetry (GUI). Accessible via the toolbar of the *FullProf Suite* under the Crystallographic Calculator button and then selecting the button Magnetic Symmetry. This application enables users to explore MSGs, view special positions, list operators, generate atomic orbits, change settings, *etc*. In Fig. 2 ▸ we can see few windows showing different options.

(2) *MHall* (console program). This second application is a command-line utility capable of generating an MSG in any setting. It computes the corresponding magnetic Hall symbol from a list of generators. An example of its use is shown in Fig. 3 ▸.

These tools provide a comprehensive framework for handling MSGs within the *FullProf Suite*, facilitating the analysis and refinement of commensurate magnetic structures.

## 6. Magnetic structure factors in *FullProf*

In conventional neutron powder and single-crystal diffraction the incident beam is unpolarized, meaning that the interference terms in the scattering cross-section do not contribute. The intensity of the Bragg reflection at **h** = **H** + **k** is simply the sum of the magnetic and nuclear contributions.
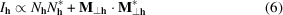
The nuclear contribution is represented by the complex nuclear structure factor *N***_h_**, which is a scalar quantity.

The magnetic interaction vector 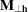 is defined in terms of the vectorial complex magnetic structure factor as
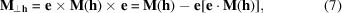
where **e** = **h**/*h* is a unit vector along the scattering vector **h**. These expressions allow the magnetic contribution to be treated as a separate *phase*, independent of the nuclear contribution. This method is particularly useful when the crystallographic structure of the paramagnetic state remains a good approximation for the magnetically ordered state. However, a complete treatment with MSG or MSSG can also be performed within a single *phase*.

A detailed description of the PCR file (input control file) is beyond the scope of this section, but in the SI we provide comprehensive documentation on the relevant variables to be used for the treatment of magnetic structures, as well as complete examples. Here we provide a brief description of these variables. A key parameter in the PCR file is Jbt, which is a phase-dependent parameter that tells *FullProf* what type of intensity calculation to perform for the current phase. The format and content of the PCR file depends on the value of Jbt. In the second section of SI we give a comprehensive summary of the different options available through the value of Jbt.

For commensurate magnetic structures, one can use the propagation vector formalism, even when a magnetic unit cell exists. The symmetry constraints can be easily obtained from the calculation of the basis vectors of the *irreps* of the propagation vector group *G***_k_**. The basis vectors can be obtained by using the program *BasIreps* (or *SARA*h**) to partially construct the PCR file if one prefers to use this method instead of MSGs. For *irreps* of dimension 1, there is a one-to-one correspondence with a MSG, and for **k** = 0 the symbol of the MSG can easily be derived from the character table of the *irreps*: the character value of −1 indicates that the corresponding operator is associated with time reversal. The basis vectors for each crystallographic site provide directly the constraints to be applied to the component of the magnetic moments. For higher-dimensional *irreps* there are several options for the possible MSGs, depending on the direction of the order parameter. Using the propagation vector formalism (Jbt=±1) and the setting Isy=-1 (see SI-2) the magnetic structure factor is calculated as
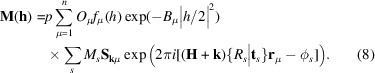
The constant *p* = ½*r*_e_γ = 0.269542 allows the conversion of Bohr magnetons into scattering lengths. The index μ runs for all magnetic atom sites in the magnetic asymmetric unit (μ = 1…*n*). The index *s* of the second sum runs over the symmetry operators described by the SYMM ({*R*_*s*_}|**t**_*s*_}, *s* = 1…*M*) and MSYM (*M*_*s*_,ϕ_*s*_) items in the PCR file. The occupation factor *O*_μ_ (distinct from *occupancy*!) is the quotient of the multiplicity of the site μ and the multiplicity of the group of operators *M*. The magnetic form factor (spherical approximation) of atom μ is *f*_μ_(*h*), with *h* = |**h**|. For simplicity, here we use the isotropic temperature factor *B*_μ_. For **k** = ½**H** the phase factors are ϕ*_s_* = 0 and the *M_s_* matrices corresponds to the MSG operators *M_s_* = δ*_s_*det(*R_s_*)*R_s_* described in the OG setting (for details, see Rodríguez-Carvajal & Bourée, 2012 ▸). In such a case, the Fourier coefficients are real and correspond directly to the magnetic moments: **S**_**k**μ_ = **m**_μ_. Alternatively, the magnetic structure factor can be expressed in terms of the basis vectors of the *irreps* as described in equation (5). The allowed coefficients 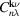 are the free parameters of the magnetic structure.

The magnetic structure factor in this case is
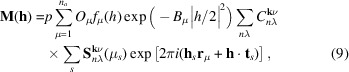
where we have written **h**_*s*_ = 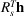 (superscript *T* stands for transpose of matrix *R_s_*). Notice that this formalism can also be applied to incommensurate magnetic structures as we will see in Section 7.

Commensurate structures have a well defined magnetic unit cell and a corresponding MSG. To describe the symmetry operators in the magnetic unit cell, one can use type-4 MSGs in the BNS setting. Various programs available on the BCS and *ISOTROPY* websites, such as *ISODISTORT* (Campbell *et al.*, 2006 ▸), can directly generate a magnetic CIF file or a PCR file template containing the appropriate list of operators. In *FullProf*, the MSG description is accessible by setting Jbt=±10, Isy=2 and Nvk=0 (see SI-2). The program can read the list of operators included in the PCR file by the user or generated by one of the programs *MAXMAGN* or *k-SUBGROUPSMAG* as a magnetic CIF file.

Additionally, the *FullProf Suite* provides a utility, *mCIF_to_PCR*, which converts a magnetic CIF file into a PCR template. This utility is accessible from the Tools menu in the *FullProf Suite* toolbar and is also available on the BCS.

The magnetic structure factor for the reflection **H**, expressed relative to the reciprocal lattice of the magnetic unit cell, relies solely on magnetic symmetry without the need for Fourier coefficients or propagation vectors:
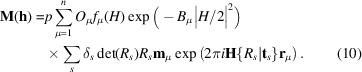
This formulation closely resembles the one used for calculating nuclear intensities in crystal structures, with the key distinction being the vectorial nature of the magnetic structure factor. Examples demonstrating the application of these options can be found in the public tutorials referenced in SI-3.

## 7. Different approaches for treating incommensurate magnetic structures in *FullProf*

### 7.1. Calculations without considering symmetry

The option Jbt=5 enables the description of a conical magnetic structure in terms of magnitudes of magnetic moments, the half-angle of the cone, the orientation of the common axis and the associate phases. This approach is particularly relevant because conical structures with a shared axis are theoretically predicted in significant classes of materials (Lyons *et al.*, 1962 ▸; Nagamiya, 1967 ▸). The formula used for computing the magnetic structure factor for both fundamental and satellite reflections follows the method established by Hasting & Corliss (1962 ▸). This option proves especially useful when dealing with incommensurate magnetic structures that exhibit macroscopic net magnetization alongside satellite reflections. In such cases, it can be effectively combined with simulated annealing, a strategy that will be discussed later.

### Calculations using **S**_**k**μ_s__Fourier coefficients or basis vectors 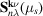
of *irreps*

7.2.

These options correspond to Jbt=±1, Nvk<0 and either Isy=-1 or Isy=-2. The structure factor expression remains formally equivalent to equations (9) and (10), but in this case the phase ϕ is not zero and the C-coefficients in equation (5) may be complex. While user can apply symmetry constraints and determine the phase factors based on the analysis of the basis vectors of the *irreps*, this process requires careful handling. Notably, in its standard application, the program *BasIreps* calculates only the representations and basis vectors of the little group *G***_k_**, from which the full representation of the star of **k** can be obtained using the induction formula.

An alternative approach allows for the use of MSYM operators, where the refinement parameters consist of simple geometrical parameters and magnetic moment magnitudes. This corresponds to the option Hel=2 (see SI-2). In this case, a specific form of the Fourier coefficients is used for the first representative of atom at site μ:
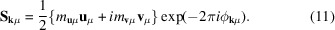
In which the unitary vectors **u**_μ_ and **v**_μ_ are orthogonal and define a plane in which the magnetic moments lay. The normal to this plane, **w**_μ_ = **u**_μ_ × **v**_μ_, completes a Cartesian frame (CF) attached to the atom. Depending of the orientation of the propagation vector **k** relative to the plane (**u**_μ_, **v**_μ_), the structure may take the form of a normal spiral or helix (if **k** is parallel to w*_μ_*) or a cycloid (if **k** is perpendicular to **w**_μ_).

Each independent atom has six free parameters: the three Euler angles defining the orientation of the CF attached to the atom relative to the crystal’s CF, the magnetic moment components m**_u_***_μ_*, m**_v_***_μ_* and the phase factor ϕ**_k_***_μ_*. If symmetry or other kind of constraints fix the CF to a particular orientation, the number of free parameters decreases. For example, if m_**u**μ_ = m_**v**μ_ the helix envelope is circular, otherwise it is elliptical. The expression for the magnetic structure factor remains identical to equation (8), with Fourier coefficients **S_k_**_μ_ replaced by equation (11).

### 7.3. Calculations using superspace operators and **T**_**k**_Fourier coefficients

This option was introduced in *FullProf* from May 2019 and corresponds to Jbt=±7 (see SI-2). Considering a general case with *d* propagation vectors **k***_p_*, a Bragg reflection is indexed using the expression
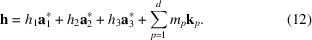
The integer indices (*h*_1_, *h*_2_, *h*_3_, *m*_1_…*m_d_*) = (*h*_1_, *h*_2_…*h*_3+*d*_) may be considered as the coordinates in the reciprocal space of a (3+*d*)D superspace. For details the reader can refer to Van Smaalen (2007 ▸). This superspace consists of a 3D section representing the real physical space (also called external space) and an internal part of dimension *d* corresponding to phase shifts of the modulation functions in the external space. In real space the modulation functions describing magnetic moments take the form of a general Fourier series:
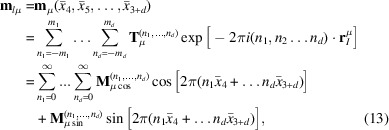
where 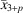 = *t_p_* + **k**_*p*_**R**_lμ_ = 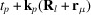 = 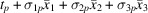 are the components of the vector 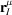 in internal space. The initial phase *t_p_* is arbitrary and can be set to zero. A point in superspace has coordinates
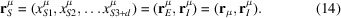
An operator 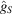 in superspace has the form
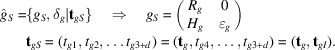
This can be written in the form of an extended matrix as
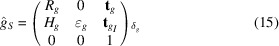
The subscript *g* refers to the operator 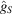. The signature parameter δ*_g_* is equal to 1 for unprimed operators and −1 for primed ones, and it is used as a multiplicative factor only when applying the operators to magnetic modulation functions. *R_g_* is the 3×3 rotational part of the parent 3D operator, *H_g_* is a *d*×3 integer matrix (with rows formed by reciprocal lattice vectors or zeroes) that may be nullified by an appropriate centred basis, and ɛ*_g_* is a *d*×*d* integer matrix with zeroes and ±1, verifying the relations: σ*R_g_* = ɛ_*g*_σ + *H_g_*, where σ is a *d*×3 matrix containing as rows the components of the propagation vectors **k**_*p*_ = (σ_1*p*_, σ_2*p*_, σ_3*p*_). These matrices are determined by the action of the 3D operators of the parent paramagnetic space group on the propagation vectors.

In the 3D crystal structure, applying the symmetry operator {*R_g_*|**t***_g_*} to an atom at site μ yields its symmetry-related counterpart μ*_g_*. If the resulting superspace operator is a symmetry of the magnetic structure, its action on the modulation function follows
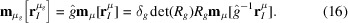
The expression of 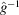 is a little bit complex, but we can write a simpler equation by substituting the argument of the transformed moment in terms of the internal coordinates of the source atom. We obtain an expression in term of operator’s items as:
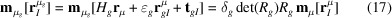
This equation is the basis for obtaining the constraints on the amplitudes 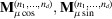 for the site μ. The program *FullProf* calculates the magnetic structure factor applying these equations when the full set of symmetry operators is derived from the provided generators. Currently, in the *FullProf Suite*, the determination of the MSSG is done by trial and error starting a process involving the group of the propagation vector and the representations. A more convenient method is to use *ISODISTORT* to generate the list of possible MSSGs and test the corresponding operators to calculate the diffraction pattern.

The key aspect is to understand how the amplitudes 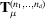 = 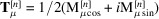 transform under superspace symmetry operators and expressing the 3D magnetic structure factor accordingly when the underlying crystal structure is unmodulated. The final formula is given by
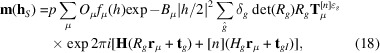
in which **h**_*S*_ = (**H**,[*n*]) are the integer indices of the reflection, *h* = |**h***_S_*| and *R_g_*, *H_g_*, ɛ*_g_***t***_g_*, and **t***_gI_* are the submatrices of the general superspace operator 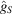 as defined in equation (15). The notation [*n*] = (*n*_1_, *n*_2_ … *n*_d_) represents a *d*-dimensional vector characterizing satellite reflections. The submatrix *ɛ_g_* transforms [*n*] into another set of indices [*m*] = [*n*]*ɛ_g_* that are equal, or opposite, to a predefined set of [*m*] and **T**^[*m*]^, and we can apply the constraint **T**^[−*m*]^ = **T**^[*m*]*^. The predefined [*m*] indices determine the harmonics of the observed satellites, denoted by the tag q_coeff in the current magnetic CIF dictionary (Campbell *et al.*, 2020 ▸). Atoms in special positions, where an operator leaves them at the same crystallographic position, impose additional constraints to their components of the magnetic moment modulation functions. These constraints can be derived from equation (17).

For NPD, *FullProf* generates all unique reflections according to the provided set of symmetry operators and the set of q_coeff [*m*], determining systematic absences and their nature, magnetic, nuclear or mixed. For NSCD, the program *nDataRed* processes raw intensity data, performing data reduction and generating a measured reflection file with superspace indices and squared magnetic interaction vectors.

## 8. Combining RA and MSG approaches for commensurate magnetic structures: symmetry modes

As discussed in Rodriguez-Carvajal & Perez-Mato (2024 ▸), the RA and MSG approaches can be used together by selecting the conventional cell basis of the MSG to define the *irreps* basis vectors. This is easily achieved through the interoperability of *FullProf* and *ISODISTORT*. Here, we describe the expression of the structure factors when using this method, referred to as the symmetry modes approach (SMA).

By adopting the cell basis of the subgroup of the paramagnetic space group and considering displacive and magnetic distortion modes from the RA, the atomic positions and the magnetic moments in the asymmetric unit can be written as
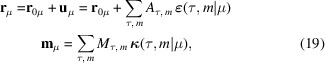
where **r**_0μ_ represents the position of the atom in the paramagnetic state, expressed in the basis of the MSG. The displacement (polar) vector **u***_μ_* is a linear combination of the different basis vectors 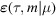 of *irrep* τ. The expression, in equation (19), for the magnetic moment follows the same structure as the equation (5), except that here the linear combination is only referred to the first representative atom of site μ without the additional *s* subscript. Furthermore, the indices *n* and λ of equation (5) are now combined in the index *m* for the basis vectors 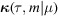. In this context, the coefficients of the linear combinations are called amplitudes of the symmetry modes. The full set of equivalent atoms in the orbit of the first representative is obtained by applying the MSG symmetry operators.

The nuclear and magnetic structure factors for reflection **H** are then expressed in terms of the amplitudes of displacive and magnetic modes (*A*_τ,*m*_, *M*_τ,*m*_) and the normalized basis vectors 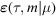 and 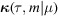 of the *irreps* (τ) contributing to the final symmetry:
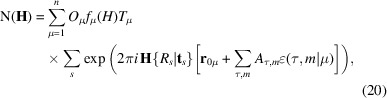

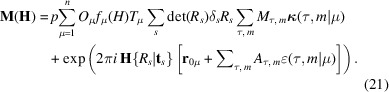


A key advantage of the SMA is that the constraints on magnetic moments and atomic positions are automatically incorporated, eliminating the need for a separate calculation. This method is implemented in *FullProf*, and *ISODISTORT* automatically generates a template PCR file for this approach. To use this option, one should set Jbt=-6 (see SI-2 and SI-3 for examples).

The SMA can also be extended to incommensurate structures using the MSSG formalism. Similar to the commensurate case, the MSSG symmetry operators can be applied as long as the modulation functions are expressed as linear combinations of the basis vectors of the *irreps* contributing to the MSSG for each particular atomic position. The Fourier coefficients 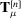 can be written as
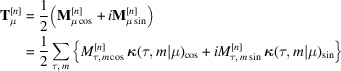
or more specifically as 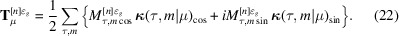
By substituting this into equation (18), the magnetic structure factor can be expressed in terms of sine, 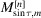, and cosine, 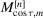, amplitudes and the precomputed constant basis vectors 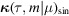 and 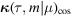. The SMA for incommensurate structures is a new development in *FullProf* and will be implemented in future versions.

## 9. Workflow for the determination of magnetic structures using the programs of the *FullProf Suite* from NPD

Here, we provide a brief overview of how to use the relevant programs within the *FullProf Suite* for magnetic structures, along with the necessary steps for determining and refining a magnetic structure using NPD.

Before determining a magnetic structure, it is essential to have a well characterized paramagnetic crystal structure, as its refinement is a prerequisite for any subsequent magnetic analysis. The process of determining a magnetic structure using NPD follows a relatively straightforward procedure, which can be summarized as follows:

(1) Collect a NPD pattern of the sample in the paramagnetic state (*T* > *T*_N_ or *T*_C_). Refine the crystal structure using the collected data to obtain all relevant structural and profile parameters. Use *FullProf* and *WinPLOTR* for this task.

(2) Collect a NPD pattern below the ordering temperature. Additional magnetic peaks typically appear in the diffraction pattern. It is important to perform a refinement of cell parameters while keeping the rest of structural parameters fixed, without including a magnetic model in the PCR file, to clearly visualize the magnetic contributions to the diffraction pattern. Determine the positions of the additional peaks simply by clicking on their positions in *WinPLOTR-2006* and saving them in a format suitable for the *k-Search* program.

(3) Determine the propagation vector(s) of the magnetic structure using *k-Search* or by trial and error, introducing an additional phase in the PCR file and treating it in Le Bail Fit (LBF) mode (without magnetic model). If no additional peaks are observed and only an extra contribution to the nuclear peaks is present, the magnetic structure has a propagation vector **k** = (0, 0, 0).

**Figure 4:**
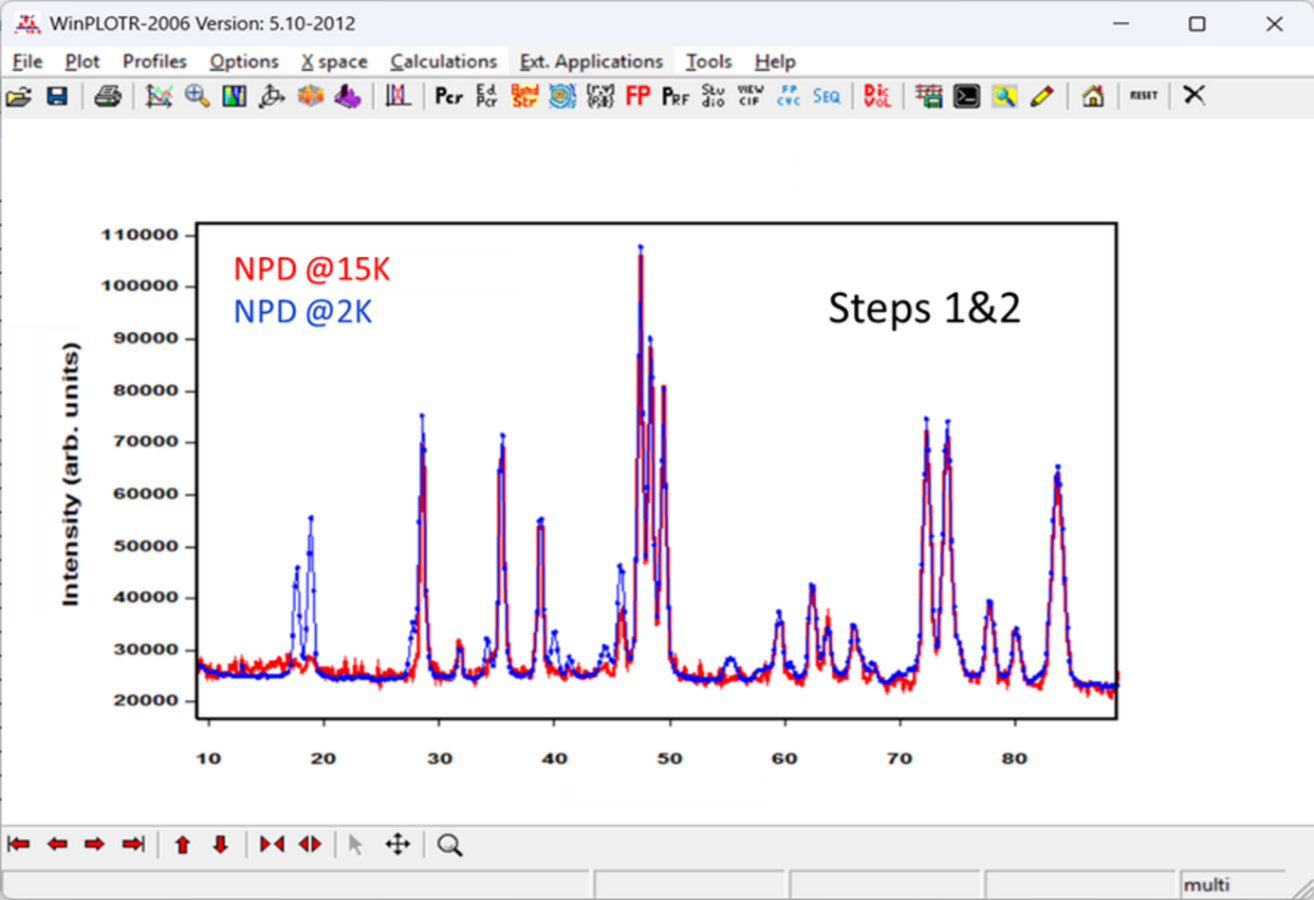
Screenshot of *WinPLOTR-2006* displaying the diffraction patterns of LiFeAs_2_O_7_ in both the paramagnetic state (red) and in the magnetically ordered state (blue), highlighting the additional magnetic reflections. This image illustrates the first two steps required to begin the analysis of magnetic NPD data.

These three steps are illustrated in the case of LiFeAs_2_O_7_ in Figs. 4 ▸, 5 ▸ and 6 ▸.

(4) Once the propagation vector has been determined, use the program *BasIreps* to obtain the basis vectors of the *irreps* of the propagation vector group *G***_k_**. For *irreps* with dimensions higher than one, the user must select the appropriate basis vectors, as *BasIreps* does not analyse the isotropy subgroups as a function of the order parameters. There are several options for selecting the appropriate symmetry of the model being constructed.

(4-i). *Commensurate structure*. Using *BasIreps*, one can determine the MSG and the corresponding magnetic symmetry operators or, alternatively, use the basis vectors of the *irreps* directly. Another approach is to use the BCS to obtain magnetic CIF files, which can then be converted into PCR file templates for possible MSGs. *ISODISTORT* also allows the direct generation of a PCR file template to work in the SMA with displacive and magnetic symmetry modes. By default, *ISODISTORT* employs a standard setting of the MSG, which may differ significantly from the parent-related setting preferred by experimentalists, but this can easily be changed before generating the template.

(4-ii) *Incommensurate structure*. The output of *BasIreps* can be used directly to construct an incommensurate magnetic structure model using basis vectors or complex Fourier coefficients. Alternatively, specific forms of magnetic structures (*e.g.* conical structures, real-space descriptions of multi-helical structures) may be employed, as described in previous sections.

(4-iii) *Incommensurate structure in superspace*. If the superspace approach is preferred, the most effective method for working with *FullProf* is to obtain magnetic CIF files from *ISODISTORT* and convert them into PCR files using the program *mCIF_to_PCR*. It is better to generate the superspace group in a setting related to the parent paramagnetic space group without changing the origin. The appropriate symbol of the superspace group can easily be determined from the symbol of the parent group (or one of its subgroups) and analysing the internal translations of the symmetry operators.

(5) Solve the magnetic structure by using the symmetry information obtained in step (4), either through trial-and-error methods or the simulated annealing (SAnn) procedure implemented in *FullProf*.

(5-i) *Trial-and-error approach*. If the RA method is to be used, modify the PCR file from step (2) by adding an additional magnetic phase, setting Jbt=1 (magnetic phase with Fourier coefficients/magnetic moments referred to the unitary basis along the unit cell axes) and Irf=-1 (only satellite reflections will be generated). The best way to add this magnetic phase is to copy it from an already existing PCR file of a similar case and adjust it using the symmetry information obtained in step (4). Run *FullProf*, keeping most parameters fixed except those for the magnetic moments or the basis functions coefficients. Check the calculated magnetic peaks against the observed data. If they do not match, modify the magnetic model (*e.g.* use a different representation or magnetic symmetry operators) and try again.

If the MSG (or MSSG) method is employed, the PCR file can be efficiently generated using *mCIF_to_PCR*, which provides a description of the model within a single phase. After generating the PCR file, modify it to include the appropriate profile parameters. Then, run *FullProf* with reasonable initial values for the moment parameters. As in previous steps, most parameters should remain fixed, with only the magnetic moment components or the cosine and sine modulation functions allowed to vary. Different MSG (or MSSG) types can then be tested against the observed diffraction data.

When the number of intense magnetic peaks is sufficiently large, this approach (using RA, MSG or MSSG) may be adequate to solve the magnetic structure. However, if the number of free parameters is too high relative to the number of observed magnetic peaks, the least-squares refinement may diverge or become unstable, especially if the initial values are far from the true minimum. In such cases, proceed to step (5-ii) for a robust optimization.

(5-ii) *Simulated annealing approach*. Modify the PCR file from step (2) by adding an additional phase in LBF mode, as in step (3). This additional phase should contain no atoms, and the settings should be Jbt=2, Irf=-1 and Jview=11. The nuclear phase should be refined with a fixed scale factor and structural parameters (except the cell parameters), allowing the separation of purely magnetic reflections into a separate file. This file can then be used by *FullProf* in SAnn mode. The details of this method are covered in the tutorials referenced in SI-3.

(6) Refine the magnetic structure using the Rietveld method implemented in *FullProf*. Once the magnetic model produces a calculated NPD pattern that closely matches the observed data, the refinement phase begins. If the trial-and-error method (5-i) was used, the refinement continues from the previous step. If the simulated annealing method (5-ii) was used, the final solution (stored in an automatically generated PCR file) must be transferred to a file suitable for refining the powder diffraction profile. However, it is also possible to continue using SAnn as a refinement procedure (see Section 10).

The specific order of the steps described above may be adjusted depending on the user’s prior knowledge of the sample.

**Figure 7:**
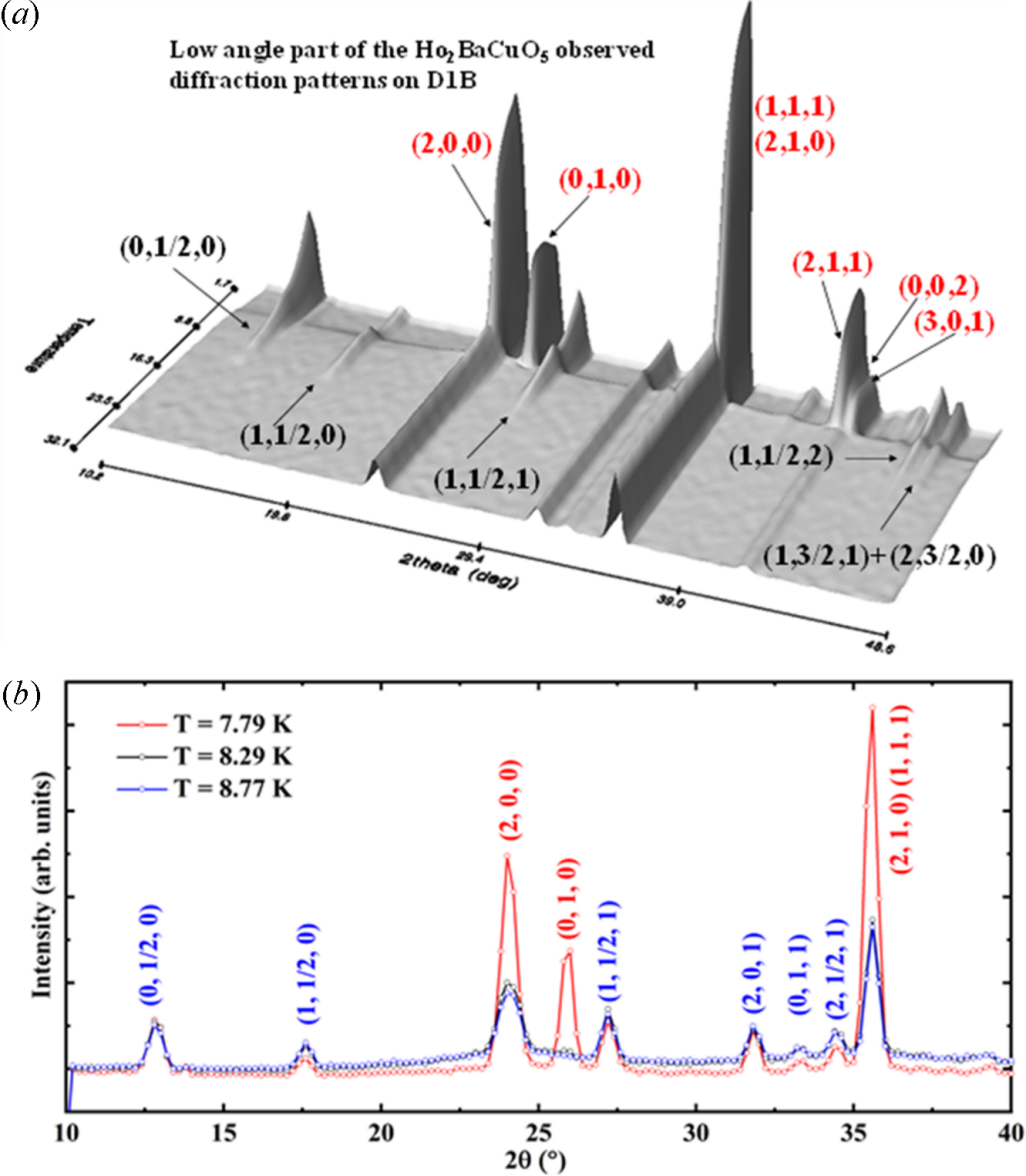
(*a*) 3D visualization of the low-angle region of diffraction patterns collected on D1B (*l* = 2.52 Å). The abrupt background change and the simultaneous emergence of the **k**_c2_ = (0, 0, 0) magnetic peaks indicates a first-order magnetic transition. (*b*) Detailed evolution of diffraction patterns for Ho_2_BaCuO_5_ around the transition at ≈8 K. The peak indexing [blue for **k**_c1_ = (0, ½, 0) and red for **k**_c2_ = (0, 0, 0)] is referenced to the paramagnetic unit cell.

Examples of the different methods for treating magnetic structures using *FullProf* are readily available in the literature. The treatment of commensurate structures using the SMA is considered the most effective approach for the reasons outlined in Rodriguez-Carvajal & Perez-Mato (2024 ▸). Examples of this method can be found in recent studies, with detailed references provided in SI-3. Here we present some results of magnetic refinements of Ho_2_BaCuO_5_ (Yanda *et al.*, 2021 ▸) as a function of temperature using the SMA, illustrated in Figs. 7 ▸ and 8 ▸.

When MSGs or MSSGs are used to analyse magnetic diffraction data, *FullProf* generates magnetic CIF files. These files can be used for data exchange and visualization purposes with external programs like *Jmol* (Jmol development team, 2016 ▸) or *VESTA* (Momma & Izumi, 2011 ▸). In other cases, such as when working with Fourier coefficients and *irreps* basis vectors, the *FullProf Suite* application *FP_Studio* enables the visualization of magnetic structures. This is achieved using .fst files, which are automatically generated by *FullProf*. Additionally, *FP_Studio* can be used interactively to learn the formalism of propagation vectors. Users can edit the .fst file directly from the GUI, manually adjusting values such as **k**, atom positions, Fourier coefficients, and more, while observing the effects of these changes in the generated picture.

## 10. Comments on least squares and simulated annealing methods in NPD

Once a reliable model for the magnetic structure is available [step (6) in Section 9], *FullProf* offers two different approaches for optimizing the agreement between the observed and calculated patterns. The conventional method is the Rietveld refinement using least-squares (LS), where *FullProf* employs the Gauss–Newton optimization algorithm. This method is fast but prone to divergence if the initial model is far from the optimal solution. Furthermore, LS is a local optimization technique, meaning it converges to the nearest minimum based on the initial model. The second approach is simulated annealing (SAnn), which is a global optimization method. For details on the algorithm applied to magnetic structure determination, refer to Rodríguez-Carvajal (1993 ▸). SAnn can be tuned for use as a refinement tool. Typically, it is used to obtain an initial model by working with clusters of integrated intensity data derived from a LBF, followed by Rietveld refinement once a reasonable model has been achieved. However, if the number of free parameters is too large, LS refinement of a magnetic structure using neutron powder diffraction (NPD) may not be effective due to inherent limitations of powder diffraction, namely, the restricted ratio of observations to degrees of freedom. This issue can be mitigated by employing SAnn with the full NPD pattern, allowing simultaneous refinement and exploration of degeneracy (*i.e.* identifying multiple possible solutions for a given diffraction pattern).

An SAnn refinement follows the same principles as a standard SAnn run, with the cost function being the reduced chi-square of the full diffraction profile and the range of variation for free parameters constrained. A greater-than-usual number of Monte Carlo cycles per temperature step is performed (typically 20 times the number of free parameters), with convergence determined by a user-defined minimal global average step. The algorithm begins with a random set of parameters within predefined boundary conditions and terminates when either the maximum number of temperature steps is reached or the average global step and chi-square change by less than 10^−4^ over two consecutive temperature steps. Unlike in least-squares refinement, the standard deviations of the refined parameters in SAnn do not carry the same statistical meaning; rather, they represent a combination of the maximum average fluctuations of moves for each parameter over the last 20 temperature cycles and a lower bound characteristic of the parameter type (typically a few per cent of the refined values).

The most effective approach is to use the difference pattern between the magnetically ordered and paramagnetic states. However, if the temperature range is too large, the difference pattern may contain numerous artefacts that hinder the refinement. In such cases, an LBF can be performed on the magnetic phase while keeping the nuclear phase parameters fixed. The calculated magnetic profile can then be saved and used subsequently for solving and refining the magnetic model.

The following steps outline how to use SAnn as a magnetic structure refinement tool in *FullProf*. To work with the full NPD profile in SAnn mode, an initial LBF run is required to generate a file with the extension .spr (hereafter referred to as the SPR file). This file contains the necessary profile parameters for subsequent SAnn refinement in *FullProf*. To generate the SPR file, the global variable Ipr must be set to -1. In this case, the SPR file is assigned the same name as the PCR file (for example, if the PCR file is named my_file.pcr, the generated SPR file will be my_file.spr). If Ipr is set to -2, the complete SPR file name must be specified on the next line of the PCR file. Additionally, Jbt must be set to Jbt=2 for LBF, along with other necessary flags. The LBF process always generates an HKL file named my_filep.hkl, or my_filep_npat.hkl (for multiple patterns), where p is the number of the phase treated with LBF and npat is the pattern number. This file contains updated values for reflection indices, multiplicities, and integrated intensities. However, the integrated intensities in the HKL file are unreliable for structure determination due to peak overlap. To generate a file that groups clusters of reflections, the variable More must be set to 1, and Jvi should be assigned one of the following values on the next line: Jvi=13 for symmetry information provided using an MSSG, or Jvi=11 for all other cases. *FullProf* then creates an INT file named my_filep_crtl.int, which contains the reflection clusters. The degree of overlap for clusters can be adjusted by modifying the default values of the RMub and RMuc parameters on the same line as Jvi (refer to the *FullProf* manual for further details).

The symmetry information for the LBF depends on the type of calculation. The variable controlling how symmetry is defined, Isy, is described in SI-2.

For Jbt=±1, when using the propagation vector formalism, setting Irf=-1 ensures that only satellite reflections are generated. After the first LBF run, Irf is automatically changed to 2, meaning that in subsequent runs the generated HKL file will be read, with stored intensities used as starting values. When working with MSGs or MSSGs (Isy=0,2), Irf should be set to 0, and it will be updated to 2 as before.

**Figure 9:**
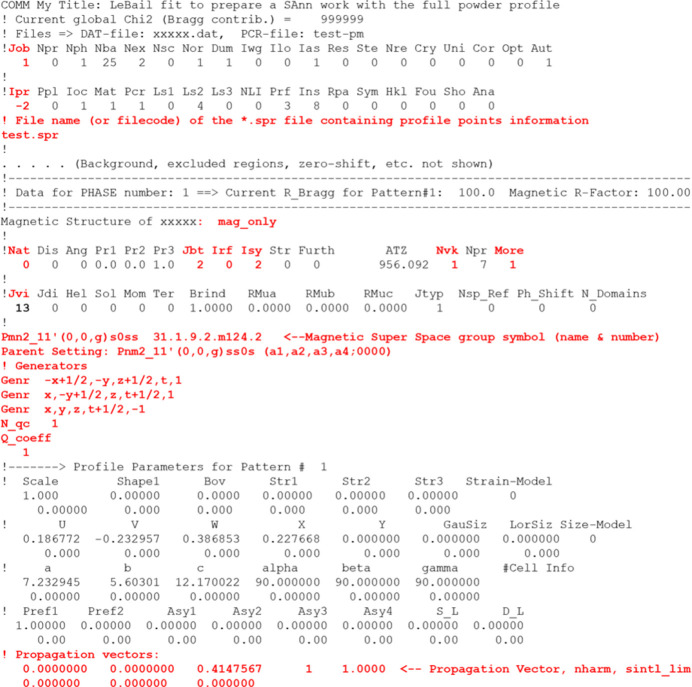
Example of PCR file (test-pm.pcr) prepared to perform an LBF on the data file xxxxx.dat. It corresponds to constant wavelength NPD data (Job=1) of and incommensurate magnetic structure described using a (3+1)D MSSG (*d* = 1) defined by its generators. The run will generate both an SPR file (Ipr=-2) of name test.spr, where the final profile information will be stored, and an INT file (More=1, Jvi=13), of name test1_ctlr.int, containing the indices of the allowed reflections (*h k l m*) according to the provided symmetry operators, and Q_coeff, multiplicity and integrated intensities. Notice that the file xxxxx.dat corresponds to a difference pattern, only the magnetic scattering is present. The keyword mag_only, appearing in the line with the name of the phase indicates to the program that it should generate only the allowed magnetic reflections.

Fig. 9 ▸ presents a template of PCR file for an incommensurate structure, demonstrating how to perform an LBF that generates the necessary files for a subsequent SAnn refinement. To prepare the PCR file for magnetic structure refinement using SAnn, the program *mCIF_to_PCR* can be used to create a general PCR template from a magnetic CIF file obtained via the BCS or *ISODISTORT*.

**Figure 10:**
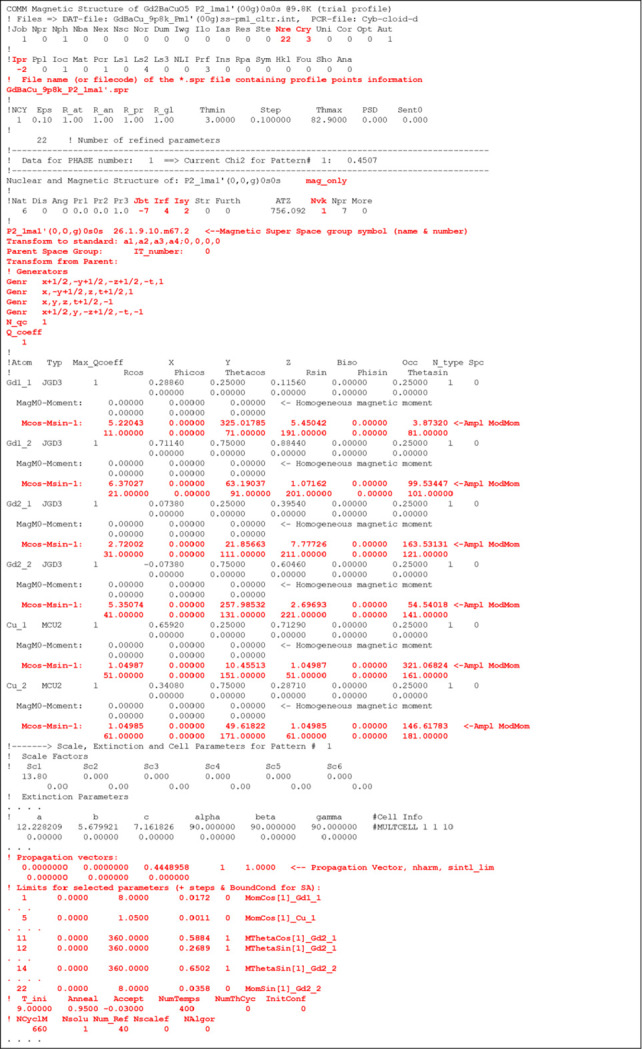
Example of PCR file prepared to perform a SAnn refinement in superspace (Cry=3, Ipr=-2, Jbt=-7, Irf=4) with modulation functions in spherical coordinates. The MSSG is generated from the provided operators. The limits of the Nre=22 parameters are quite large, so a global search is applied. The value of the parameter Accept means that the, in the last stages of the algorithm, it acts as a refinement procedure.

**Figure 11:**
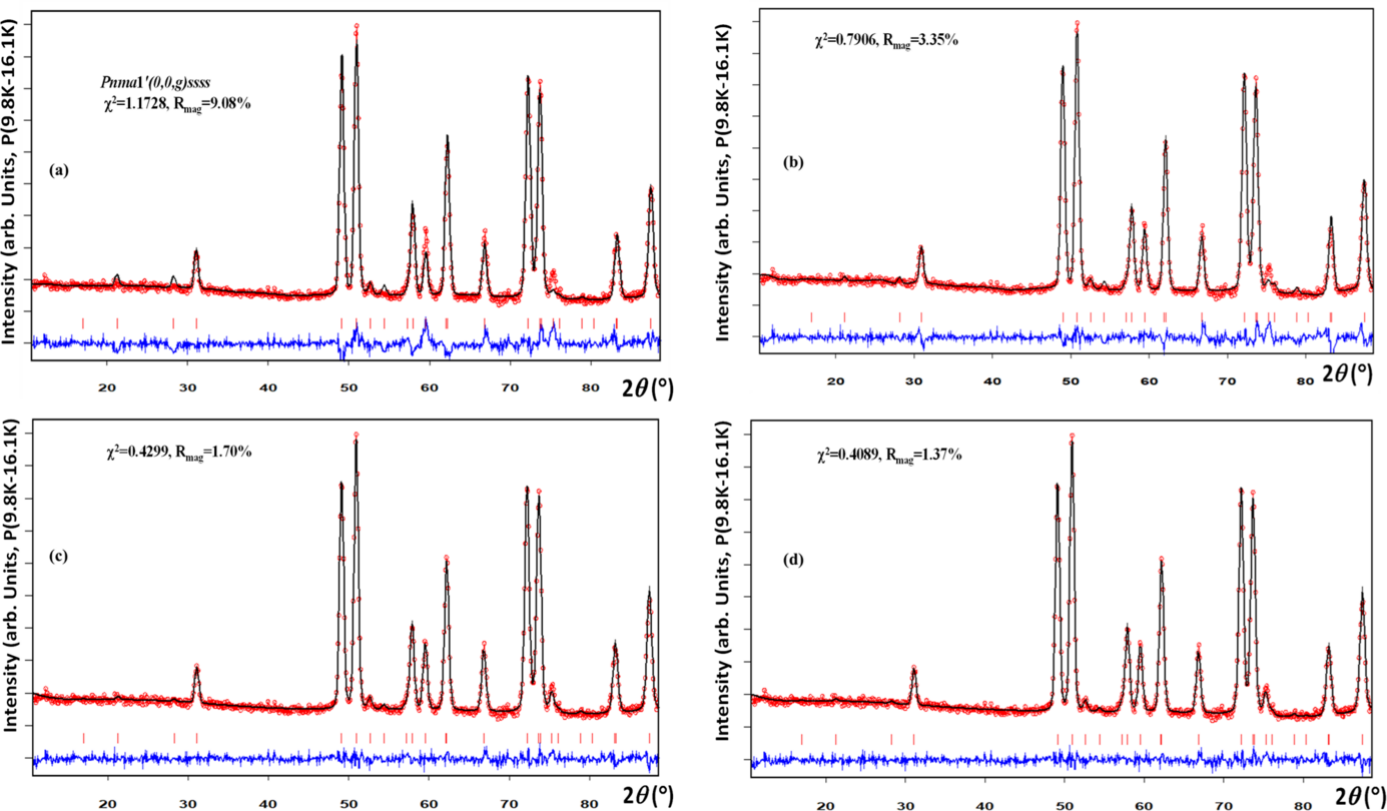
Plots of the different SAnn refinements of the difference pattern (9.8 K–16.1 K) for four models of Gd_2_BaCuO_5_ magnetic structure. (*a*) *Pnma*1′(0,0,*g*)*ssss*, three sites, 12 free parameters. (*b*) *Pnm*2_1_1′(0,0,*g*)*ss*0*s*, six sites, 23 free parameters. (*c*) *P*2_1_*ma*1′(0,0,*g*)0*s*0*s*, six sites, 22 free parameters, using the same amplitudes for the Cu atoms. This corresponds to the mixing **irreps* mLD*2⊕*mLD*3 for the order parameter *P-P*(*a*,0|*b*,0) in the notation of *ISODISTORT*. The PCR file is that of Fig. 10 ▸. (*d*) *Pm*1′(0,0,*g*)*ss*, 12 sites and 32 free parameters. We concluded that the (*c*) case is the true magnetic structure of Gd_2_BaCuO_5_, because the subgroup in (*d*) has too many parameters and the improvement is negligible. For details consult the text and the SI of Yanda *et al.* (2020 ▸).

An example of the application of SAnn to the NPD difference pattern is provided in the literature for the multiferroic compound Gd_2_BaCuO_5_. More details can be found in the published work by Yanda *et al.* (2020 ▸) and the SI of this paper. In summary, Gd_2_BaCuO_5_ undergoes two magnetic phase transitions. Upon cooling, long-range magnetic ordering occurs at *T*_N_ = 11.8 K with a propagation vector **k** = (0, 0, γ) followed by a lock-in transition to a strongly non-collinear structure at *T*_loc_ ∼ 6 K, with **k**_c_ = (0, 0, 1/2). The paramagnetic MSG is P*nma*.1′ and, after evaluating the possible MSSGs using *ISODISTORT*, it became evident that the information contained in the NPD difference pattern was insufficient for performing a full Rietveld refinement of the possible models, as refinements allowing all degrees of freedom proved unstable. As described in previous sections, SPR and INT files were generated for the different symmetries, and SAnn jobs were executed using scripts invoking the console version of *FullProf*. Fig. 10 ▸ presents key sections of the PCR file used to analyse the data at 9.8 K for one of these cases. Fig. 11 ▸ compares the refined profiles obtained for different MSSGs.

## 11. Conclusion

We have demonstrated how *FullProf* uses different formulations of magnetic structure factors to model magnetic neutron diffraction data. Additionally, we have provided a detailed, step-by-step guide for working with NPD, illustrating the procedure with examples that may be particularly useful for those new to magnetic structure determination. The SI includes access to tutorials containing data files and PCR files, showcasing various examples in which the workflow and different refinement options discussed in the text are applied.

We hope this paper, together with the SI, will serve as a valuable resource for researchers in condensed matter physics or chemistry seeking to understand the exotic properties of materials through magnetic structure determination.

## Supplementary Material

Sections SI-1, SI-2 and SI-3. DOI: 10.1107/S2052520625003944/cam5008sup1.pdf

## Figures and Tables

**Figure 5:**
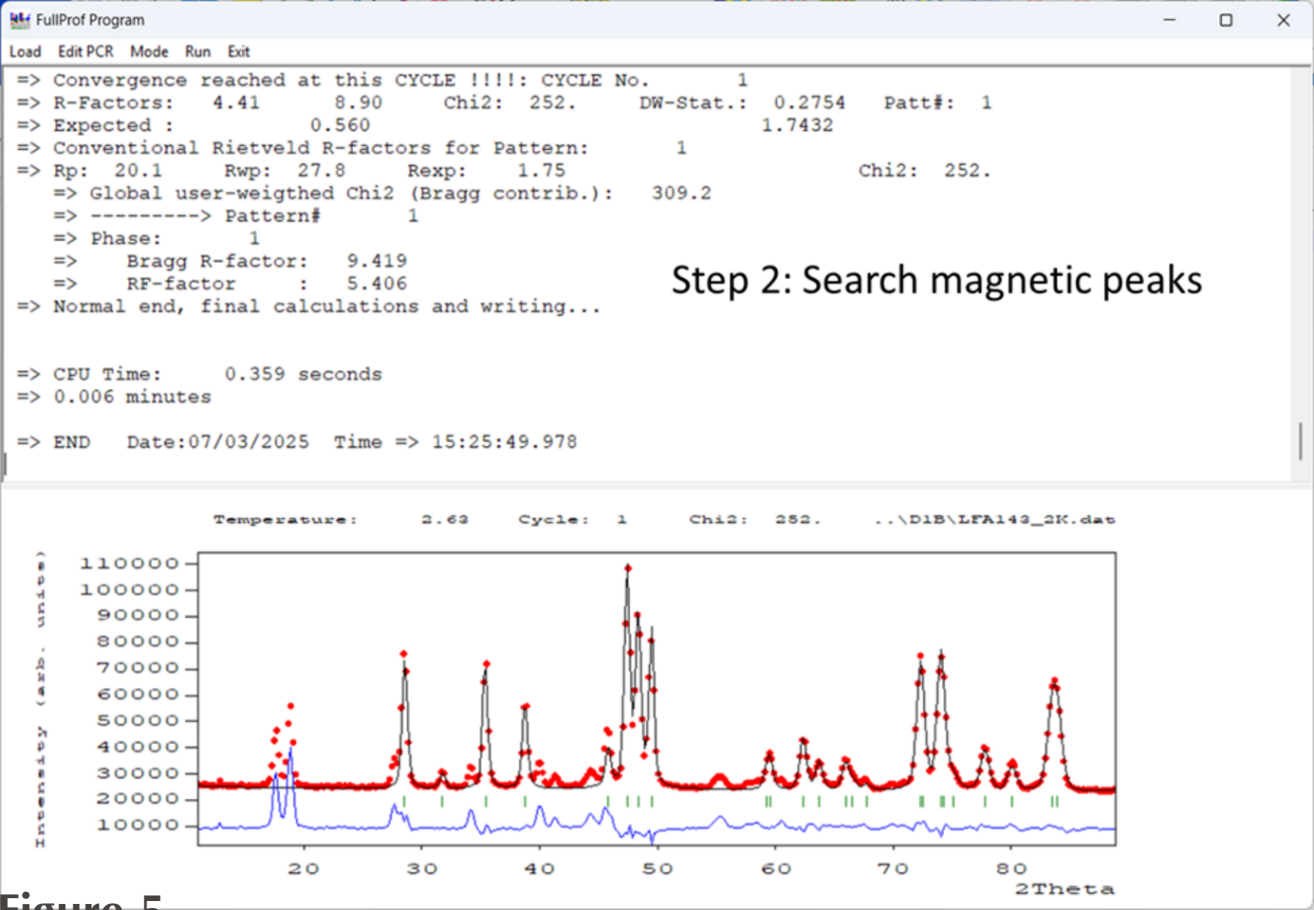
Execution of *FullProf* without a magnetic model, where the paramagnetic structure is fixed along with the linearly interpolated background. Only the scale factor, unit-cell parameters, and zero shift are refined. This process generates a profile file with corrected angular positions, enabling further analysis in *WinPLOTR-2006* to determine the propagation vector.

**Figure 6:**
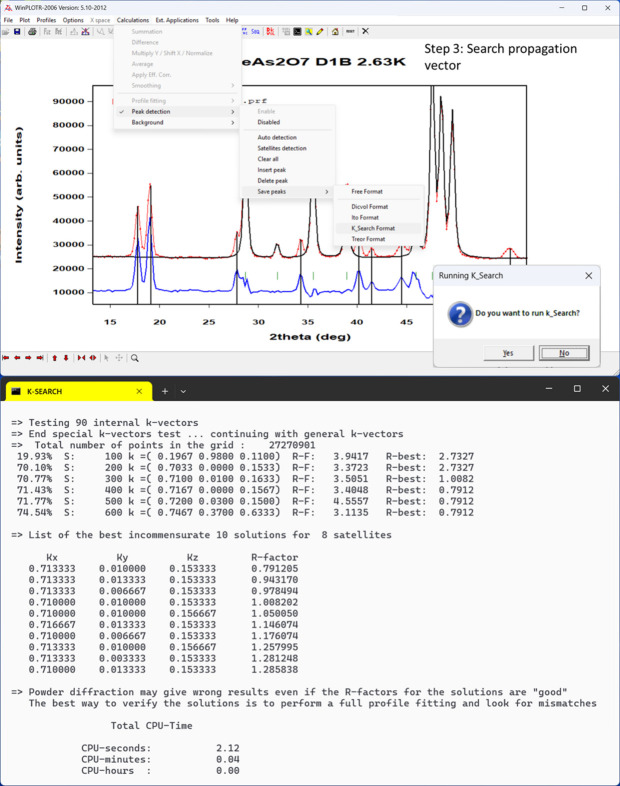
Selection of magnetic peak positions in *WinPLOTR-2006*, generating an intermediate file that stores cell parameters and user-defined conditions from a previous dialogue. Peak positions are automatically corrected for zero-shift. Upon exiting the dialogue, the program prompts the user to run *k-Search*; if accepted, it proceeds using the prepared file. The lower section of the figure displays the identified solution, with the resulting **k**-vector**k** ≈ (0.713, 0.010, 0.153).

**Figure 8:**
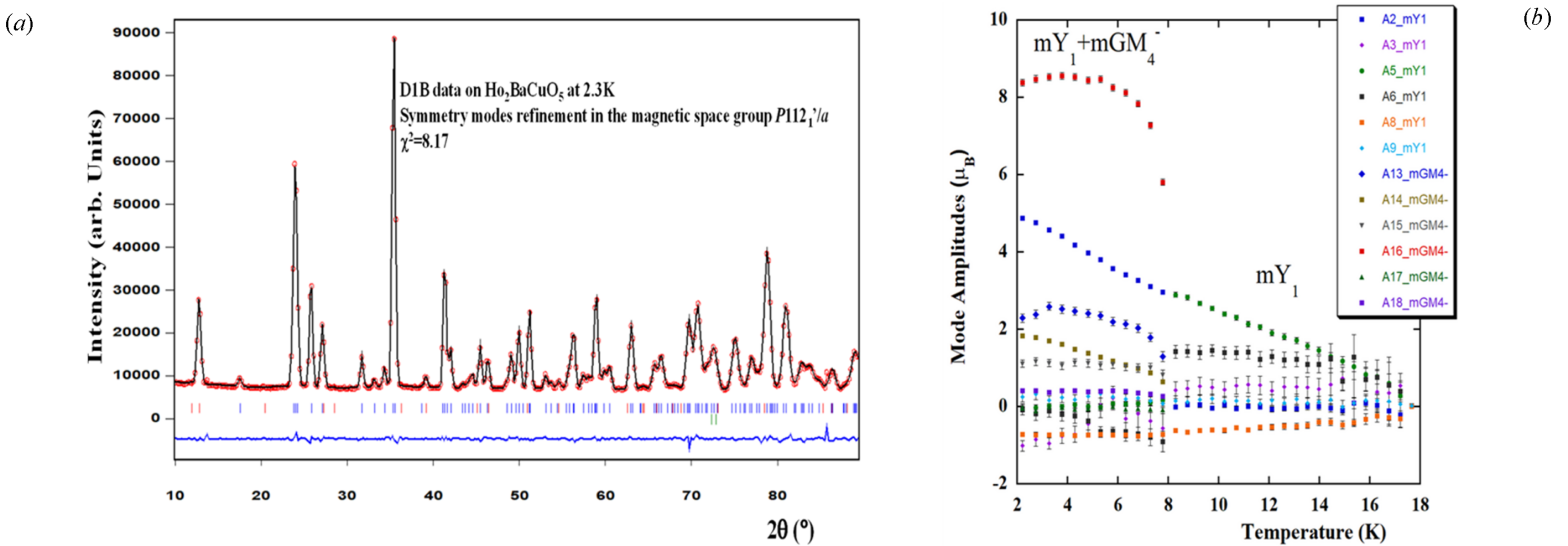
(*a*) Refinement plot of the lowest-temperature NPD pattern of Ho_2_BaCuO_5_ in the MSG, using symmetry modes amplitudes. (*b*) Temperature dependence of the magnetic amplitudes of the symmetry modes. For details, refer to the SI of Yanda *et al.* (2021 ▸).
