# Supplementary material for: Magnetic structure determination and refinement using FullProf
Source: Acta Crystallogr B Struct Sci Cryst Eng Mater. 2025 May 28;81(Pt 3):302–17. doi: 10.1107/S2052520625003944 (PMC12147938; doi:10.1107/S2052520625003944)
Supplement: Supplementary file 1 [file b-81-00302-sup1.pdf]

Acta Cryst B

STRUCTURAL SCIENCE
CRYSTAL ENGINEERING
MATERIALS

Volume 81 (2025)

Supporting information for article:

Magnetic structure determination and refinement using *FullProf*

Juan Rodriguez-Carvajal, Javier Gonzalez-Platas and Nebil A. Katcho

Supplementary Information for:

Magnetic structure determination and refinement using FULLPROF

Juan Rodríguez-Carvajal*[a], Javier González-Platas[b] and Nebil A. Katcho[a]

[a] Diffraction and Computer for Science Groups of the Institut Laue-Langevin, 71, Avenue des Martyrs, CS 20156, 38042 Grenoble Cedex 9, France.

[b] Departamento de Física (IUDEA), Universidad de La Laguna, Avenida Astrofísico Francisco Sánchez s/n, La Laguna, Tenerife E-38204, Spain.

*email: jrc@ill.eu

SI-1: General Features of the PCR files

The input control file required by FULLPROF is a text file of moderate complexity, partially in free format. Not all items in a line need to be present, but all lines must be provided, except in certain cases where the previously selected options do not require them. There are specific rules for constructing these files, all of which are detailed in the FULLPROF manual and in the document `fp2k.inf`.
Manually creating a PCR from scratch using a text editor can be quite complex and error-prone, often leading to program malfunctions. The most efficient approach is to use one of the many example files included in the FULLPROF SUITE distribution and modify it according to the user's requirements. Another option is to use the CIFs_to_PCR utility, which can generate a wide variety of PCR files from a given instrumental resolution function (IRF) file, data files, and CIF files (see Panel 1). For handling magnetic data, the mCIF_to_PCR utility converts magnetic CIF files into PCR templates (see Panel 2).

A PCR file contains comment lines —denoted by an exclamation mark (!)—that are ignored by the program. These comments provide short names for parameters and flags that indicate actions or information for the program. Beneath each parameter/flag name, numerical values define different options. Parameters designated for refinement are accompanied by refinement codes, known as *code-words*.

Code-words may appear on the same line as the parameter —for example, in the case of the zero-shift for Constant Wavelength (CW) diffraction—or on the line below the parameter value. A code-word follows the format of a real number: `"xxxm.abc"` where

"xxx" is an integer number representing the parameter's ordering number: its position in

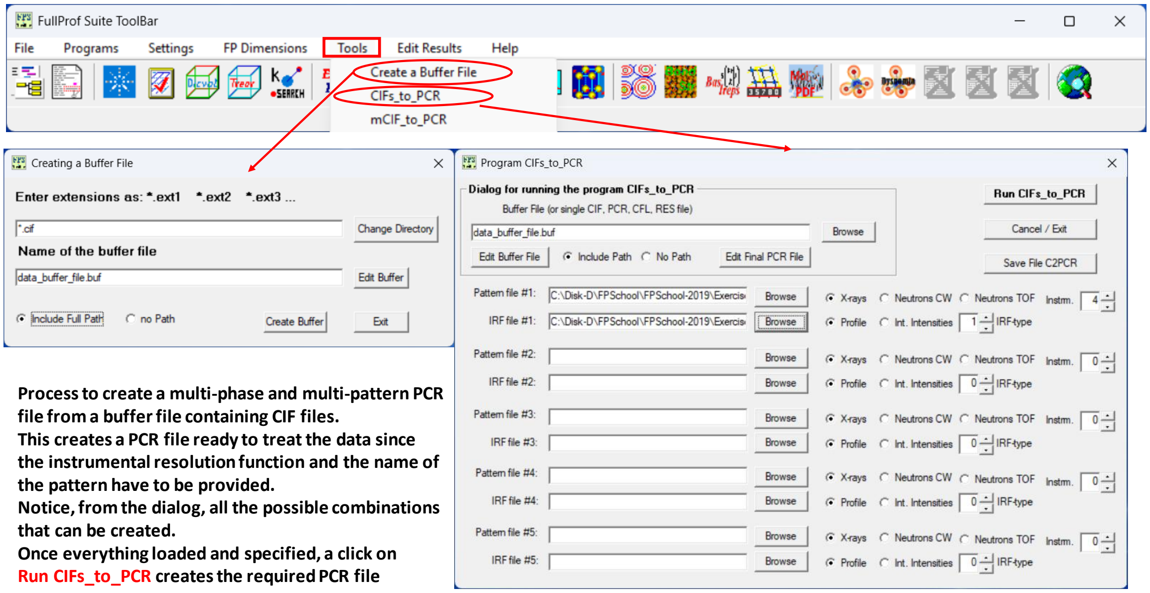

Panel 1: This image shows how to create a buffer file containing CIF files and a PCR file from the created buffer file. To use this application IRF files are compulsory.

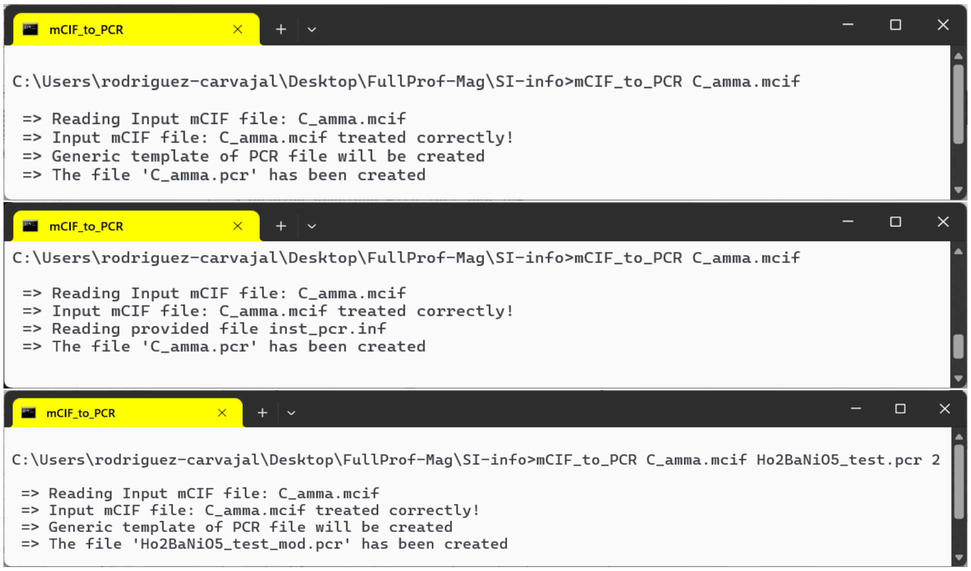

Panel 2: Examples of running `mCIF_to_PCR` from the command line. Loading a mCIF file into the toolbar and selecting `mCIF_to_PCR` from the `Tools` menu (see Panel 1) generates a generic PCR file that the user must adapt manually. If there exist a file of name **`inst_pcr.inf`** in the current directory, the appropriate wavelength, instrument, background and U, V, W parameters are used instead of creating a generic template. The different options for running MCIF_TO_PCR from the command line are displayed in this panel. See note of 16 August 2024 in file `fp2k.inf` for details.

rows or columns within the least-squares (LS) matrix, and "m.abc" is a multiplier applied

to the calculated shift at each refinement cycle. This multiplier is typically set to 1.00, which is the expected value in the Gauss-Newton optimisation method. However, the user may diminish this value to prevent divergence in complex cases. Code-words also enable straightforward parameter constraints: if two different parameters share the same code-word, FULLPROF applies a common calculated shift—after inverting the LS matrix—to update both parameter values in the next LS cycle. Manually assigning code-words can be tedious; for this reason, the **`Aut`** flag (see SI-2) can be set to 1. In this case, instead of specifying a code-word, the user simply enters 1.0, indicating that the parameter should be refined. FULLPROF then automatically assigns an appropriate code-word with a multiplier of 1.0. The user can later fine-tune the multipliers as needed.
Another important feature to be aware of is the availability of specific *keywords* (commands) that can be used in place of directly managing code-words. For this functionality, the **`Aut`** flag must be set to 1. Currently, there are both global commands and phase- and pattern-dependent commands. A list of the most relevant phase- and pattern-dependent keywords is available in the document `FP_COMMANDS.pdf`. This document is accessible from the FULLPROF SUITE toolbar under the Help menu: Help > Manuals > PDF files > FP_Commands.

The most critical section of a PCR file is the one defining the *phases* contributing to the various patterns being analysed. This section contains both the *structural parameters* and *profile parameters* for each pattern. Examples of PCR files are presented in Figures 9 and 10 of the main text. In this context, a *phase* refers to the description of a *crystal or magnetic structure (or both)* for which the program will perform a specific type of calculation, contributing to the total calculated pattern(s). The type of calculation performed depends on the values assigned to various flags. As this discussion focuses on magnetic structures, the list of relevant flags is provided in Section SI-2.

SI-2: Relevant Flags for Magnetic Structure Determination in the PCR file

In the PCR file contains several *flags* that may be *global* (applicable to the entire file) or *pattern-dependent* (specific to each diffraction pattern). These flags play a crucial role in all FULLPROF options.

Global Flags

`Nph` –Number of phases present in the PCR file
`Nre` –Number of boundary conditions to be used. This is mandatory for simulated annealing and corresponds to the number of free parameters.
`Aut` –If set to 1, code-words are assigned automatically.
`NLI` –Number of linear restraints applied. These restraints can involve various parameters across patterns and phases.

Pattern-Dependent Flags

The most general pattern-dependent flags relevant to our purposes are:

`Job`– Controls the type of calculation and the radiation used. Acceptable values for each pattern are:

- **`Job= 0`** – CW X-ray diffraction (refinement or structure solution).
- **`Job= 1`** – CW neutron diffraction (refinement or structure solution).
- **`Job= 2`** – CW X-ray diffraction (pattern simulation).
- **`Job= 3`** – CW neutron diffraction (pattern simulation).
- **`Job=-1`** – TOF neutron diffraction (refinement or structure solution).
- **`Job=-3`** – TOF neutron diffraction (pattern simulation).
- **`Job= 4`** – Electron diffraction (only single-crystal data).

`Cry`– Specifies single-crystal/powder calculations. In the *single-pattern* format of the PCR file, this is a *global* indicator. For *multiple-patterns*, it is replaced by **`Int`** (for integrated intensities) that takes a different value for each pattern. The different values acceptable for each pattern are:

- **`Cry= 0`** – Powder pattern.
- **`Cry= 1`** – Single crystal (twinned or not) or powder integrated intensities.
- **`Cry= 2`** – Monte Carlo search with integrated intensities.
- **`Cry= 3`** – Simulated Annealing (only single pattern and phase can be processed).
- **`Cry= 4`** – Simulation of single crystal patterns.

`Uni`– Defines the type of scattering variable used. Acceptable values for each pattern are:

- **`Uni= 0`** – Scattering variable is **2θ** in degrees.
- **`Uni= 1`** – Scattering variable is **TOF** in micro-seconds.
- **`Uni= 2`** – Scattering variable is **Energy** in keV.

Phase-dependent flags

The flag **`Jbt`** determines how the contribution of the current phase to the diffraction pattern(s) is calculated. Some cases also require additional flags (**`Irf`**, **`Isy`**, **`Hel`**, **`Nvk`**) to specify particular options. These apply to both crystal and magnetic structures. Below is a summary of the available **`Jbt`** options, focusing mainly on magnetic structure analysis in FULLPROF. For further details, consult the manual or the news file (**`fp2k.inf`**) accessible from the toolbar. The possible values of **`Jbt`** are:

- **`Jbt= 0`** – Phase treated as pure crystallographic (no magnetism). This is the standard option for the Rietveld method for neutrons and X-rays.
- **`Jbt= 1`** – Standard approach for magnetic structures using propagation vectors formalism. Only the magnetic contribution is calculated. The refinable parameters are the components of vectors $\mathbf{R}_{\mathbf{k}\mu}$ and $\mathbf{I}_{\mathbf{k}\mu}$ (Equation 3) or the coefficients $C_{n\lambda}^{\mathbf{k}\nu}$ of the basis vectors of irreducible representations.
- **`Jbt=-1`** – As above, but the components of the Fourier coefficients $\mathbf{R}_{\mathbf{k}\mu}$ and $\mathbf{I}_{\mathbf{k}\mu}$ are given in spherical coordinates. Refinable parameters include the modulus and the spherical angles of these vectors.

Jbt=±2 – Le Bail fit mode with a constant arbitrary scale factor. Integrated intensities are treated as free parameters obtained by an iterative procedure. Between each iteration, LS cycles are applied to profile parameters. The integrated intensities (**Jbt=2**) or the structure factor's modules (**Jbt=-2**) of reflections are output to a reflection file.

Jbt=±3 – The intensity (**Jbt=3**) or the structure factors (**Jbt=-3**) are read from a file and kept fixed. Only the scale factor and profile parameters are refined.

Jbt=±4 – Pure crystallographic phase where rigid body blocks are defined (refer to the manual/tutorials for details).

Jbt= 5 – *P*1 real space description of incommensurate *conical magnetic structures*, with an arbitrary cone axis orientation.

Jbt= 6 – The crystallographic contribution is treated using *symmetry modes* from a higher-symmetry phase. This is typically generated by BCS using AMPLIMODES.

Jbt=-6 – Nuclear and magnetic contributions are treated in a single phase using *symmetry modes* from a higher symmetry phase (e.g. the paramagnetic phase). Only for *commensurate* structures (generated by ISODISTORT).

Jbt=±7 – Magnetic structure described using magnetic superspace groups (MSSGs). The negative sign indicates that the modulation functions $\mathbf{M}_{\mu\cos}^{[n]}$ and $\mathbf{M}_{\mu\sin}^{[n]}$ are given in spherical coordinates. Only incommensurate magnetic structures within a commensurate crystal structure are currently supported. This format for PCR files is generated by mCIF_to_PCR when the magnetic CIF corresponds to an incommensurate magnetic structure described using an MSSG.

Jbt=±10– Nuclear and magnetic phases are combined into a single phase, where either MSGs or standard Fourier coefficients (as is **Jbt=±1**) can be used. The negative sign indicates that spherical coordinates are used for magnetic moments.

Additional Phase-Dependent Flags for Magnetic Structures

Nvk – Specifies the number of propagation vectors defining the magnetic structure. If **k** is not equivalent to -**k**, `Nvk` takes a negative value, except for **Jbt=±7**, where **Nvk** is always positive as it refers to the number of pairs (**k**,-**k**).

Irf – Determines how reflections are generated:

Irf=0 – Generated from the space group symbol or from a list of symmetry operators.

Irf=2 – Indices and multiplicities are read from a file.

Irf=-1 – Only satellite reflections are generated.

Irf=4 – Experimental integrated intensities are provided for the given phase (only for single-pattern format of the PCR file).

Isy – Defines how symmetry is handled in the magnetic phase:

`Isy= 0`– Symmetry operators are generated automatically from the space group symbol or read from the database of Shubnikov groups. In the latter case other considerations should be complied that are discussed in the examples and tutorials.

`Isy= 1` – Symmetry operators provided as numerical matrices and vectors (obsolete).

`Isy=-1`– Symmetry operators are provided in symbolic form as SYMM/MSYM (e.g. `SYMM x,-y+1/2,z; MSYM -u,v,-w,`ϕ). See discussion below Equation (8).

`Isy=-2`– SYMM operators are used for atomic positions, while Fourier components are expressed as linear combinations of *irreps* basis vectors.

`Isy= 2`– SYMM symbols include now +1 (non-primed operators) or -1 (primed operators), e.g. `x,y+1/2,-z,-1`.
For superspace approach only the generators of the group are needed in the form: `x,y+1/2,-z,u+1/2,-1` or as described in magnetic CIF files as `x1,-x2,x3,-x4+1/2,1`.

`Hel=2:` When used in combination with **`Jbt=-1`** (or **`Jbt=-10`**) and **`Isy=-1`**, the program applies the direct-space formulation of multi-helical incommensurate magnetic structures described in the text (see Equation 11).

SI-3: Tutorials, links and references

Recently, we have uploaded a series of tutorials and exercises on magnetic structures to the FULLPROF SUITE website (https://www.ill.eu/sites/fullprof/php/tutorials.html). The relevant tutorials, under the header **Magnetic Structures**, are numbered from 1 to 9. Below, we will outline the content of these tutorials and exercises.

Tutorial 1: This is an introductory-level tutorial based on the magnetic structure of $LaMnO_3$. The magnetic structure is characterised by the propagation vector **k**=(0,0,0) with respect to the paramagnetic group P*bnm*.1′, making it is the simplest of all the cases covered. Two diffraction patterns, above and below the Néel temperature (300K and 50K). The user is guided step by step through the process of solving and refining the magnetic structure. This includes using EDPCR to create PCR files, BASIREPS and the BCS for applying the RA and MSG approaches, respectively. The detailed steps are outlined in the document `Tutorial_LaMnO3.pdf`.

Tutorial 2: This tutorial represents a further step in complexity and is based on the magnetic structure of Ho_2BaNiO_5. Only the low temperature diffraction pattern is provided, but the crystal structure (space group I*mmm*) is well-known. The preparation of the PCR files is performed using EDPCR and some text editing by hand. The program K-SEARCH is used to determine **k**. The magnetic structure is characterised by the propagation vector **k**=(1/2,0,1/2), and its star has two arms. The single-**k** is analysed using both BASIREPS and

the BCS, while the multi-**k** option is explored using the program of the BCS k-SUBGROUPMAG. It is demonstrated that similar agreement between observed and calculated patterns is achieved for both single-**k** and multi-**k** solutions. However, physical considerations lead to the preference of the single-**k** case. All the steps followed are detailed in the document `Tutorial_Ho2BaNiO5.pdf`. Additionally, an annex has been included in the document to explain how to construct MSYM operators from the output of BASIREPS, as well as some useful tips for handling cases where the *irreps* have a dimension greater than 1.

Tutorial 3: This corresponds to a series of exercises for determining the magnetic structure of KTb_3F_{12}. The paramagnetic space group is I4/*mmm*.1′ and the propagation vector is **k** = (1, 1, 1). Representation analysis, simulated annealing and symmetry modes are used for solving and refining the magnetic structure. The exercise using the RA-method illustrates the case in which 1D complex representations must be combined into 2D representations in order to get real Fourier coefficients. This is because **k** is equivalent to -**k** and the imaginary Fourier coefficients should be zero as they represent directly magnetic moments.

Tutorial 4: This tutorial serves as an introduction to using FULLPROF for analysing magnetically ordered single crystals. The example selected is a single crystal of the compound $Cs_2FeCl_5{\cdot}D_2O$. Reflection intensities were collected at the D9 diffractometer (ILL) with λ=0.841 Å. A complementary NPD pattern was measured at D1B. All data are provided. A detailed explanation of the subject can be found in *Phys. Rev.* B **96**, 104428 (2017), DOI: 10.1103/PhysRevB.96.104428
The magnetic structure is solved using BASIREPS, and the user is guided for all steps by combination of EDPCR and a text editor. Everything is explained in the document `Tutorial_Cs2FeCl5·D2O.pdf`.

Tutorial 5: This tutorial demonstrates how to work with single crystals in FULLPROF using a structural model described in the PCR file in terms of two phases. In this particular case, the multiphase approach is employed to facilitate the refinement of a magnetic structure characterised by the propagation vector **k**=(0,0,0). The magnetic space group (MSG) splits the atoms in such a way that the full set of new degrees of freedom cannot be properly refined due to the weakness of the magneto-elastic coupling. The structural and magnetic description of $Na(OH_2)_3]Mn(NCS)_3$ can be found in the manuscript by *Geers et al. Phys. Chem. Chem. Phys.*, **26**, 15844 (2024) https://doi.org/10.1039/d4cp01265h.
In the document `Tutorial_Multiphase_SXtal.pdf`, all steps are explained. The data reduction program DATARED is used to process the data at 25K in the paramagnetic state. A console program, working on Windows, modifies the intensity file generated by DATARED to format it appropriately for multiphase treatment of single crystals.
Everything is detailed in the document `Tutorial_Multiphase_SXtal.pdf`.

Tutorial 6: This tutorial outlines the use of FULLPROF for analysing the commensurate magnetic structure of $DyFeWO_6$ using the Symmetry Modes Approach (SMA). The data used in this tutorial were published in the reference provided below [1]. The tutorial illustrates that the single *irrep* of the space group *Pna*2_1, for **k**=(0,1/2,1/2) is two-dimensional an complex when using the standard option in the program BASIREPS. The use

of k-SUBGROUPSMAG, which provides mCIF files that can be easily converted to PCR files, offers a simpler alternative to manually deducing how to combine the basis vectors. This is a typical example where the SMA proves to be straightforward. ISODISTORT can directly generate the PCR file in the correct format. All the steps are detailed in the document: `Tutorial_DyFeWO6.pdf`.

Tutorial 7: This tutorial is similar to Tutorial 6 but focus only on providing data and tips for applying different methods. It includes NPD data as a function of temperature for Ho_2BaCuO_5 collected using the CW data from the old D1B instrument (ILL), as well as TOF data from WISH (ISIS) at a fixed temperature. The SMA can be applied to sequential refinements, which is the case in this tutorial. A data buffer file containing the names of the D1B data files (with relative paths) is provided and can be used with WINPLOTR-2006 to visualise the entire set of NPD data in 3D, highlighting the phase transitions. PCR files and a script (`sequential_HoBaCuO.bat`), compatible with Windows but easily adaptable for Linux or MacOS), are provided and ready to run from a terminal. For further details, refer to the document `Tutorial_Ho2BaCuO5.pdf`.

Tutorial 8: This addresses a simple incommensurate magnetic structure occurring in $LiFeAs_2O_7$. The structure contains a single magnetic atom, Fe, located at the origin, forming a simple Bravais lattice. The data were published in *Physical Review* **B 88**, 214433 (2013). This paper, along with the document `Tutorial_LiFeAs2O7.pdf`, can be found in the root directory of the tutorial. NPD data collected above and below the Néel temperature, are provided for practical exercises. The tutorial document guides the user step-by-step through solving the magnetic structure using different methods: BASIREPS, special real space methods (**`Jbt=5`**) and (**`Jbt=-1`**, **`Hel=2`**, **`Isy=-1`**), and MSSG using ISODISTORT to generate magnetic CIF files, which are then converted to PCR files by using MCIF_TO_PCR. The various methods for treating incommensurate magnetic structure are compared.

Tutorial 9: This tutorial explores a more complex incommensurate magnetic structure in $DyMn_6Ge_6$. Only low temperature data are provided, and the crystal structure is assumed to be known. The magnetic ordering involves two propagation vectors: **k**=(0,0,0), responsible for a macroscopic net magnetisation, and **k**=(0,0,δ), δ≈0.165, responsible of the incommensurability. In this tutorial the magnetic structure of the compound is solved using ISODISTORT to construct different MSSGs, which are then converted to PCR files. The user is guided through the steps in the document `Tutorial_DyMn6Ge6.pdf`.

Published papers with adequate Supplementary Information for learning the use of the FULLPROF SUITE interoperating with the BCS or with ISODISTORT

There are three papers (two of them have been referenced in the main text) containing sufficient information in their Supplemental Material (SM) for learning about the use of the FULLPROF SUITE. We provide here the complete references and links to their SM.

[1] *Ordered aeschynite-type polar magnets* RFeWO$_6$ (R=Dy, Eu, Tb, *and* Y): *A new family of type-II multiferroics*
Somnath Ghara, Emmanuelle Suard, François Fauth, T. Thao Tran, P. Shiv Halasyamani, Akira Iyo, Juan Rodríguez-Carvajal, and A. Sundaresan
Phys. Rev. B 95, 224416 – Published 13 June, 2017
DOI: https://doi.org/10.1103/PhysRevB.95.224416
https://journals.aps.org/prb/abstract/10.1103/PhysRevB.95.224416#supplemental

[2] *Interplay of* $4f-3d$ *interactions and spin-induced ferroelectricity in the green phase* Gd$_2$BaCuO$_5$
Premakumar Yanda, I. V. Golosovsky, I. Mirebeau, N. V. Ter-Oganessian, Juan Rodríguez-Carvajal, and A. Sundaresan
Phys. Rev. Research 2, 023271 – Published 3 June, 2020
Erratum Phys. Rev. Research 2, 049002 (2020)
DOI: https://doi.org/10.1103/PhysRevResearch.2.023271
https://journals.aps.org/prresearch/abstract/10.1103/PhysRevResearch.2.023271#supplemental

[3] *Magnetic-field-induced ferroelectric states in centrosymmetric* R_2BaCuO$_5$ (R=Dy *and* Ho)
Premakumar Yanda, F. Orlandi, P. Manuel, N. Boudjada, J. Rodriguez-Carvajal, and A. Sundaresan
Phys. Rev. B 104, 144401 – Published 1 October, 2021
Erratum Phys. Rev. B 105, 099901 (2022)
DOI: https://doi.org/10.1103/PhysRevB.104.144401
https://journals.aps.org/prb/abstract/10.1103/PhysRevB.104.144401#supplemental
